# Urine-based diagnostic tests for tuberculosis: a scoping review highlighting unmet diagnostic needs

**DOI:** 10.3389/fmicb.2026.1783312

**Published:** 2026-03-27

**Authors:** Mayu Hikone, Yuya Kondo, Yu Takaizumi, Wataru Kagaya, Katharina Kranzer, Nobuo Saito

**Affiliations:** 1 Kenya Research Station, Institute of Tropical Medicine, Nagasaki University, Nagasaki, Japan; 2 School of Medicine, Nagasaki University, Nagasaki, Japan; 3 Department of Ecoepidemiology and Epidemiological Informatics in Tropical Medicine, Nagasaki University Graduate School of Biomedical Sciences, Nagasaki University, Nagasaki, Japan; 4 Department of Ecoepidemiology, Institute of Tropical Medicine, Nagasaki University, Nagasaki, Japan; 5 Clinical Research Department, London School of Hygiene and Tropical Medicine, London, United Kingdom

**Keywords:** biomarker, concentration, diagnosis, transrenal DNA, tuberculosis, urine

## Abstract

**Introduction:**

Current diagnostics for tuberculosis (TB) rely on sputum or site-specific specimen collection, posing critical limitations in certain populations. Urine has emerged as an alternative specimen, as demonstrated by urine lipoarabinomannan (LAM) testing. Beyond LAM, no urine-based tests are established for clinical use, and evidence for non-LAM urine antigen tests and urine-based nucleic acid amplification tests (NAATs) remains inconsistently synthesized. This scoping review aimed to comprehensively map current evidence and identify gaps in urine-based diagnostic tests for TB.

**Methods:**

We conducted a scoping review across three databases (PubMed, Scopus, and Embase) of studies evaluating urine-based diagnostic tests for TB and reporting diagnostic performance metrics, extracting data on urine test type, country, TB disease type, study population, and HIV status. Findings were summarized descriptively and visualized to illustrate trends. We also extracted technical approaches for urine-based NAATs and urine concentration methods.

**Results:**

In total, 208 publications reporting 274 urine-based tests published between 1995 and 2024 were included. Most studies were conducted in high-burden settings and focused on pulmonary TB (44.7%), adult populations (62.5%), and people living with HIV (31.3%). LAM-based tests dominated the literature, accounting for 49.3% (*n* = 135) of all tests, primarily in lateral flow formats. Non-LAM urine antigen tests were evaluated less frequently (*n* = 26), typically in small cohorts and laboratory-based assays. Urine-based NAATs showed wide variability in technical approaches. Only a few studies (*n* = 10) evaluated urine concentration methods.

**Conclusion:**

Evidence for urine-based diagnostic tests for TB remains limited for extrapulmonary TB, children, and non-HIV individuals, despite substantial unmet need. Although urine LAM is the most established assay, evaluations of non-LAM urine antigen tests and urine-based NAATs remain exploratory, with few studies, small cohorts, and predominantly laboratory-based assays. Further research is needed to identify and validate reliable, broadly applicable urine-based diagnostic tests to address these gaps.

## Introduction

A rapid and accurate diagnosis of tuberculosis (TB) is essential for timely treatment, improved outcomes, and reduced transmission. However, an estimated 2.4 million individuals with TB remain undiagnosed annually (World Health Organization, n.d.). Current diagnostic tools have limitations. Culture-based detection of *Mycobacterium tuberculosis* (MTB), while considered the gold standard, is slow, resource-intensive, and often inaccessible in resource-limited settings where TB burden is high. Although acid-fast bacilli (AFB) smear microscopy is widely available, it lacks sensitivity.

Sputum-based diagnosis has long been the mainstay of TB testing. Nucleic acid amplification tests (NAATs) performed on sputum, such as the Xpert MTB/RIF (Cepheid, Sunnyvale, CA, United States) and Trunat (Molbio Diagnostics, Goa, India) assays, have substantially expanded access to rapid and sensitive diagnostics and drug-resistance testing. However, increasing challenges have emerged regarding patient groups in whom sputum is difficult to obtain or insufficient for reliable testing. People living with HIV (PLWH), young children, and critically ill patients may struggle to expectorate sputum (Peter et al., 2012; Thomas, 2017) or produce samples with low bacillary loads, resulting in reduced test sensitivity (Hargreaves et al., 2001). Furthermore, for patients with extrapulmonary TB (EPTB), diagnosis typically requires site-specific specimens such as cerebrospinal fluid, pleural fluid, or tissue obtained through biopsy. These procedures are often invasive and may be unavailable at the district level or in resource-limited healthcare facilities. Additionally, the performance of current NAATs remains suboptimal for these non-sputum specimens (Kohli et al., 2021). A central challenge in TB diagnosis, therefore, is the reliance on sputum and site-specific specimens.

Given these limitations, diagnostics based on non-site-specific specimens, particularly urine, have emerged as an alternative. Urine collection is simple, safe, and well-suited for patients who are unable to produce sputum. Most studies have focused on urine-based lateral flow assays (LFAs) to detect lipoarabinomannan (LAM), a cell wall component of MTB. Although the World Health Organization (WHO) has recommended urine LAM testing for PLWH, its diagnostic utility is largely confined with advanced immunosuppression and is further limited by concerns about specificity (Broger et al., 2023; Nathavitharana et al., 2021). In addition to LAM, urine contains circulating microbial components such as short fragments of cell-free DNA, often referred to as transrenal DNA (trDNA), which may offer high specificity and broader diagnostic potential (MacLean et al., 2021; Green et al., 2009). Nevertheless, despite these promising characteristics, urine-based diagnostic tests for TB remain underutilized, and further investigations are needed to determine their full clinical value.

Although these advances highlight the growing expectations and promise of urine-based diagnostic tests for TB, existing evidence remains fragmented. Most existing reviews have focused primarily on urine LAM tests (Broger et al., 2023; Nathavitharana et al., 2021; Seid et al., 2022; Sood et al., 2023; Chatla et al., 2021; Minion et al., 2011; Gupta-Wright et al., 2016; Bjerrum et al., 2019; Shah et al., 2016) and urine-based NAATs (mainly the Xpert MTB/RIF assay) in the context of urogenital TB (UGTB; Altez-Fernandez et al., 2017; Chen et al., 2020). The biological bases of urine diagnostics differ according to disease type. In UGTB, intact MTB bacilli may be directly shed into urine, enabling organism-based detection using urine-based NAATs or culture. In contrast, in non-UGTB disease, diagnostic targets must originate from MTB components that pass the glomerular filtration barrier, such as antigens or trDNA released from degraded organisms (Peter et al., 2010). These different mechanisms have important implications for assay design and performance. However, the broader landscape of urine-based diagnostic tests remains poorly characterized. Previous reviews have emphasized test accuracy, with limited attention to technical approaches such as urine pretreatment (e.g., concentration methods), which markedly influence test sensitivity (Connelly et al., 2021; Lawn et al., 2009; Savolainen et al., 2013; Lawn et al., 2017; Kerkhoff et al., 2017). Furthermore, technical variations (e.g., DNA extraction, target genes, and amplicon design) in urine-based NAATs used to detect MTB trDNA have not been described in the current literature, which may impact overall test performance (Green et al., 2009; Oreskovic et al., 2021).

Therefore, a scoping review is needed to map the current landscape of urine-based diagnostic tests for TB, identify knowledge gaps, and guide future research. Specifically, the objective of this review was to synthesize evidence across urine-based approaches, with particular attention to technical approaches that may affect test performance. Ultimately, we aimed to highlight both the potential and limitations of urine-based diagnostic tests and inform future diagnostic developments.

## Methods

The protocol was informed by the Joanna Briggs Institute methodology for scoping reviews (Aromataris et al., n.d.), the Preferred Reporting Items for Systematic Reviews and Meta-Analysis Protocols (PRISMA-P; Moher et al., 2015), and PRISMA Extension for Scoping Reviews (PRISMA-ScR; Tricco et al., 2018), and registered prospectively with the Open Science Framework on July 29, 2024.^1^ The review was performed in accordance with PRISMA-ScR standards (Supplementary material 1). Patients or the public were not involved in the design, or conduct, or reporting, or dissemination plans of our research.

### Data source and search strategy

We systematically searched three electronic databases, MEDLINE (via PubMed), Scopus, and Embase, from their inception through July 2024. The search was limited to studies published in English involving human samples. English-language and human-study filters were applied during the search stage in Embase, these filters were not applied in MEDLINE or Scopus. In MEDLINE, animal-only studies were excluded using the standard syntax “NOT (animals[MESH] NOT humans[MESH])”, while in Scopus, language and population restrictions were applied during the title/abstract and full-text screening stages. We performed the final database search on July 30, 2024. Our primary search terms included “urine,” “diagnosis,” and “tuberculosis.” We adapted the search strategy for each database by incorporating relevant keywords and index terms to maximize sensitivity. The full search strategy for each database is shown in Supplementary material 2.

### Eligibility criteria

We applied the population-concept-context (PCC) framework to define the scope of this review and included studies that evaluated the diagnostic performance of urine-based diagnostic tests for active TB. To capture the overall research trends, we included studies that enrolled individuals with any form of TB, including PTB and EPTB, and did not exclude UGTB at the review level.

Although our primary conceptual focus was urine as a non-site-specific specimen, where diagnostic targets originate from MTB components that pass the glomerular filtration barrier, such as antigens or trDNA, complete exclusion of UGTB at the search and screening stage was not feasible. Some studies enrolled mixed EPTB cohorts that included UGTB without stratified reporting by disease site. Excluding such studies at the inclusion stage would have risk of introducing bias. Accordingly, UGTB was not excluded during initial study selection and was included to describe the overall study characteristics and trend. However, for targeted syntheses focusing on diagnostic strategies aligned with the non-site-specific urine paradigm, particularly urine-based NAATs, studies exclusively addressing UGTB were excluded to maintain biological and conceptual coherence.

We accepted reference standards that relied on microbiological confirmation, such as culture or NAATs (e.g., Xpert MTB/RIF assay) that directly detected MTB from affected sites, or composite reference definitions that combined microbiological, clinical, radiological, or histological evidence, as described by the study authors. To ensure broad applicability, the studies were not restricted by age group or comorbidities (e.g., PLWH). However, studies that evaluated latent rather than active MTB infections were excluded. This review included urine-based diagnostic tests, encompassing tests designed to detect MTB DNA, pathogen-derived antigens (e.g., LAM), or host-derived markers in human urine samples. Given the exploratory nature of several diagnostic studies, we imposed no restrictions on the study context, thereby capturing evidence from diverse healthcare settings and environments.

We included published journal articles written in English that reported at least one diagnostic performance metric, such as positivity rate, sensitivity, specificity, or area under the receiver operating characteristic curve (AUC). We accepted a variety of study designs, including experimental, quasi-experimental, and observational studies (prospective and retrospective cohort, case–control, and cross-sectional studies).

We excluded studies that fell outside the PCC framework or met one or more of the following criteria: qualitative studies; narrative or systematic reviews; case reports or case series; opinion papers; conference abstracts, gray literature, or conference proceedings; studies using non-human or synthetic urine samples; spike-in experiments; studies published in languages other than English; studies with fewer than 20 urine samples; and studies that relied solely on secondary data analysis, such as cost-effectiveness models or machine learning approaches without primary diagnostic data. Studies for which full-text access was unavailable were also excluded.

### Screening process

To ensure consistency, all reviewers (MH, YK, YT, WK, and NS) screened an initial set of 50 publications, discussed the results, and revised the screening and data extraction manual before proceeding with a full review. All retrieved articles were compiled and uploaded to Rayyan (Rayyan Systems Inc., Doha, Qatar) for duplicate removal. Two reviewers (MH, YK, YT, or WK) independently screened the titles and abstracts, followed by full-text screening. We documented the reasons for exclusion at the full-text stage. A third reviewer (NS) resolved any disagreements regarding article selection.

### Data charting process and data items

One author (MH) extracted data from the included studies in a standardized manner. Two reviewers (YK and YT) cross-checked the extracted information, and any discrepancies were discussed until a consensus was reached. The following data were extracted from each included study: author, publication year, age group (adults or children), TB disease type (PTB, EPTB, or UGTB), country where the study cohort was recruited, HIV status, urine-based diagnostic test evaluated, total sample size, reference standard used for TB diagnosis, and diagnostic performance of the test. For descriptive mapping purposes, the unit of analysis differed according to the type of summary. Study-level characteristics (e.g., population and setting) were summarized at the level of the publication. In contrast, analyses of diagnostic approaches were conducted at the level of the individual urine-based index test. When a single study evaluated more than one urine-based assay, each index test was counted separately in the assay-based summaries, while remaining linked to the same underlying study cohort. For pathogen-based tests (i.e., antigen and DNA), we extracted details on the urine volume used for analysis and the concentration methods applied. Centrifugation alone was not considered a concentration method in this review, as it was most often performed as part of decontamination procedures (e.g., NALC-NaOH and Petroff’s method) or for debris removal. For urine-based NAATs, we applied an additional criterion: studies focused on UGTB were excluded from the urine-based NAAT synthesis because our conceptual focus was to characterize NAAT performance when urine was used as a non-site-specific specimen. For urine-based NAATs, we additionally extracted information on sample storage conditions, collection timing, urine pretreatment methods (e.g., preservatives, centrifugation, and DNA extraction methods), target genes, and amplicon sizes.

### Synthesis of results

We synthesized the findings using descriptive summaries and visualizations to map the landscape of urine-based diagnostic tests for TB. Study-level characteristics were summarized using the number of included publications as the denominator unless otherwise specified. Analyses of diagnostic approaches were conducted at the level of individual urine-based index tests. Key study characteristics, such as diagnostic target, test platform, analytical methods, study population, and clinical context, were extracted into an overall summary table. To characterize temporal trends in research activity, we generated a bubble chart plotting each study by publication year and test type. Bubble size reflected the number of studies published in each category-year combination, allowing visualization of shifts in research focus. Additionally, to characterize the breadth of diagnostic strategies, we developed a network-style bubble diagram illustrating the distribution of pathogen- and host-derived tests. The bubble size was proportional to the number of studies within each diagnostic target category, allowing comparison of evidence density across domains. Moreover, we prepared detailed summary tables describing the urine-based NAAT workflow and urine concentration methods.

## Results

### Study selection and characteristics

**Figure 1:**
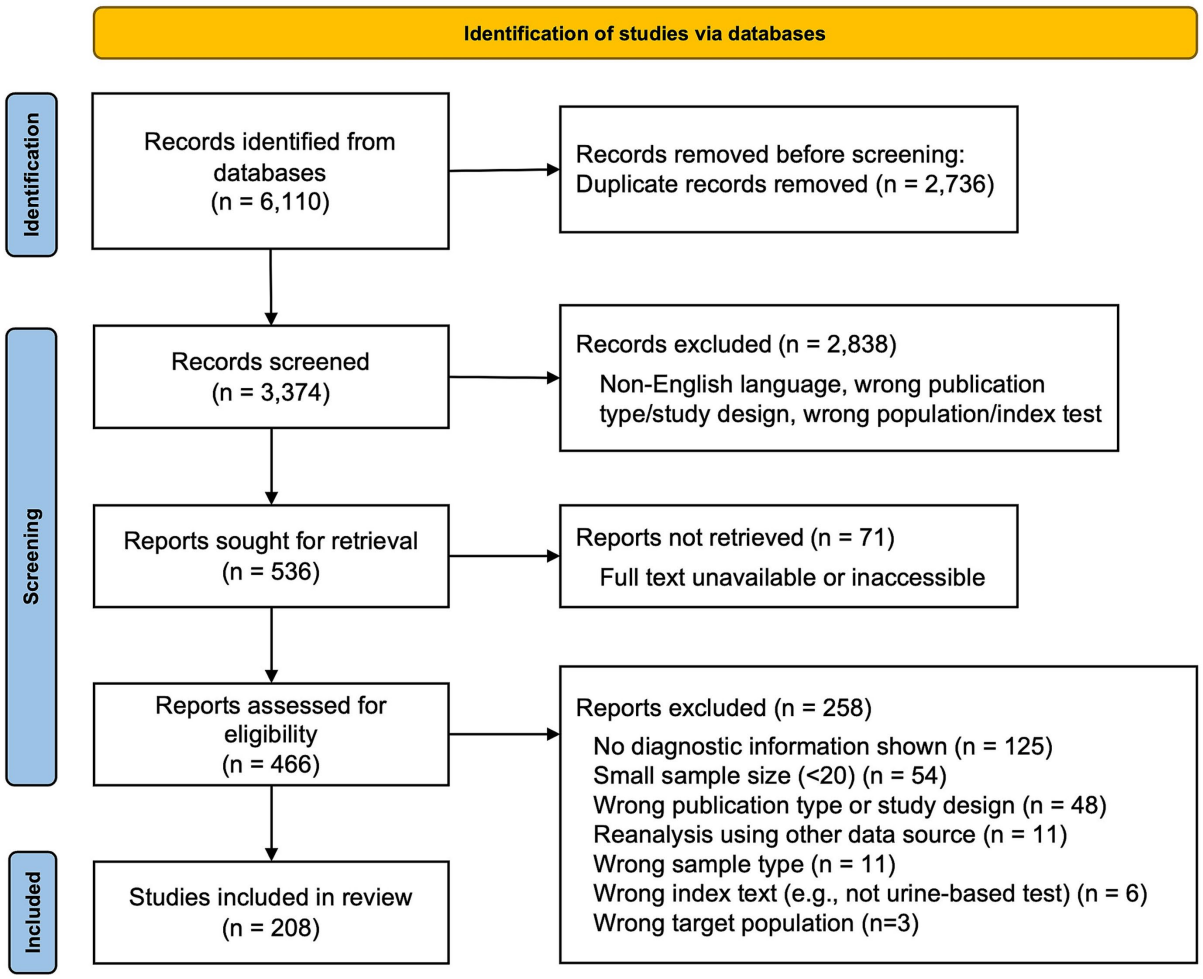
PRISMA flow diagram. This diagram illustrates the number of records identified, screened, excluded, and included in the scoping review. The records were retrieved through a database search, followed by duplicate removal, title/abstract screening, and full-text assessment. The final number of studies included in the review is shown at the bottom of the flow.

The database search identified 6,110 records related to urine-based diagnostic tests for TB (Figure 1). After removing 2,736 duplicate records, 3,374 unique records were screened by title and abstract for relevance to the review objectives. Of these, 71 publications were not retrieved, mainly due to unavailable full texts of older publications, leaving 466 publications for full-text assessment. After full-text screening, 258 publications were excluded because they contained no diagnostic information (*n* = 125), had small sample sizes (<20; *n* = 54), used ineligible publication types or study designs (*n* = 48), involved reanalysis using secondary data sources (*n* = 11), used non-human or inappropriate sample types (*n* = 11), assessed non-urine-based tests (*n* = 6), or targeted populations outside the study scope (*n* = 3). In total, 208 publications met the inclusion criteria and were included in the final review (Supplementary material 3). These studies were published between 1995 and 2024 and conducted in 57 countries. Among the 208 studies, 32 reported adherence to the Standards for Reporting of Diagnostic Accuracy Studies (STARD) guideline (Bossuyt et al., 2015).

### Population and settings

Table 1 summarizes the study characteristics. Geographically, the highest number of studies were conducted in Africa (52.2%, *n* = 129/247), followed by Asia (29.6%, *n* = 73/247) and South America (7.3%, *n* = 18/247). Studies were predominantly conducted in South Africa (*n* = 57), followed by Uganda, India, China, and Tanzania. The majority of the study cohort was recruited from high-TB burden countries as defined by the WHO (60.7%, *n* = 150/247).

**Table 1:** Study characteristics (*n* = 208).

Characteristics	Studies, *n* (%)
Country (*n* = 247)	
Africa	129 (52.2)
Asia	73 (29.6)
South America	18 (7.3)
Europe	16 (6.5)
North America	11 (4.5)
Disease type	
PTB	93 (44.7)
PTB and EPTB	55 (26.4)
EPTB	20 (9.6)
UGTB	16 (7.7)
TB disease type not clearly reported	24 (11.5)
Age	
Adult	130 (62.5)
Children	20 (9.6)
Adult and children	7 (3.4)
Not reported	51 (24.5)
HIV status	
PLWH	65 (31.3)
Both PLWH and non-HIV individuals	59 (28.4)
Non-HIV individuals	24 (11.5)
Not reported	60 (28.8)

PTB; pulmonary tuberculosis; EPTB, extrapulmonary tuberculosis; UGTB, urogenital tuberculosis; PLWH, people living with HIV. Percentages may not total 100% due to rounding.

Regarding TB disease type, studies predominantly focused on PTB, either alone or in combination with EPTB. Specifically, 44.7% (*n* = 93/208) of the studies exclusively targeted PTB, whereas 26.4% (*n* = 55/208) included both PTB and EPTB. Studies on EPTB without UGTB as the primary focus accounted for 9.6% (*n* = 20/208), whereas UGTB-specific studies accounted for 7.7% (*n* = 16/208). The type of TB was not clearly reported in 11.5% (*n* = 24/208) of studies.

Most studies included in this review (62.5%, *n* = 130/208) focused on adult populations, whereas only 9.6% (*n* = 20/208) targeted pediatric populations. A small proportion of the studies (3.4%, *n* = 7/208) targeted populations across all age groups, including adults and children. Among the included studies, 31.3% (*n* = 65/208) focused exclusively on PLWH, 28.4% (*n* = 59/208) included both PLWH and non-HIV individuals, and 11.5% (*n* = 24/208) included only non-HIV individuals. HIV status was not clearly reported in 28.8% (*n* = 60/208) of studies.

### Overview of urine-based diagnostic tests

**Figure 2:**
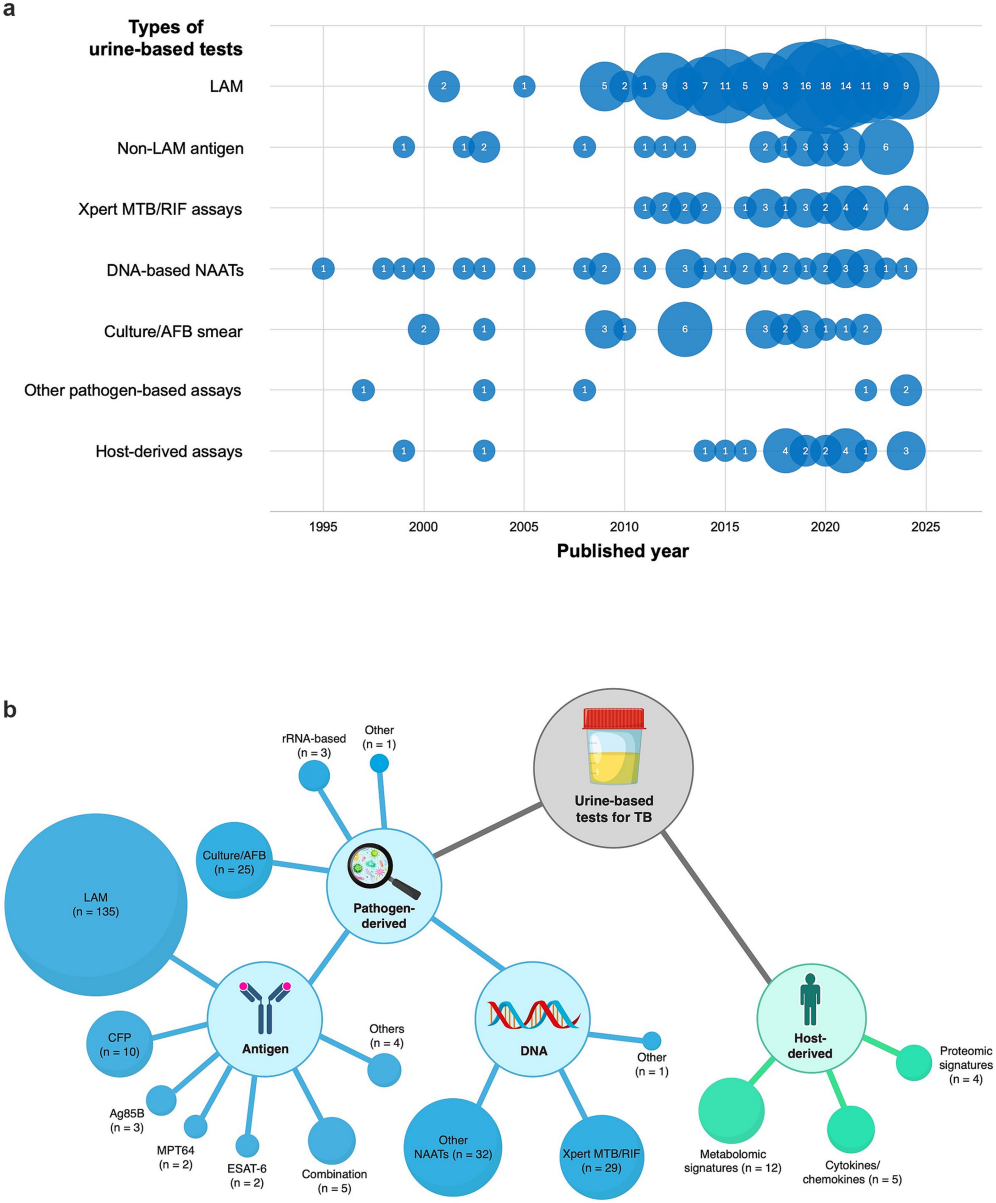
Bubble charts for urine-based diagnostic tests for tuberculosis (TB). **(a)** Temporal distribution of studies (1995–2024) by category. **(b)** Network of pathogen- and host-derived assays. Bubble size reflects the number of studies. TB, tuberculosis; AFB, acid-fast bacilli; LAM, lipoarabinomannan; CFP, culture filtrate protein; ESAT-6, early secreted antigenic target of 6 kDa; NAATs, nucleic acid amplification tests.

In total, 208 studies evaluated 274 urine-based diagnostic tests. The temporal distribution of urine-based TB diagnostic studies by test type is illustrated in Figure 2a, and the overall distribution of the test type is shown in Figure 2b. The number of publications increased steadily since the early 2000s, with 71.2% (*n* = 195/274) of the tests published in the past 10 years.

LAM-based tests were the most frequently investigated, with sustained publication activity since 2009 and accounting for 49.3% (*n* = 135/274) of all included tests. Non-LAM urine antigens (e.g., CFP, MPT64, ESAT-6, and Ag85B) demonstrated considerably lower publication intensity (9.5%, *n* = 26/274). The earliest reports appeared sporadically in the late 1990s and early 2000s, with a slight increase in the past 5 years, but without a surge comparable to that of LAM.

Studies focused on the urine-based molecular assays were the second most frequent, accounting for 24.1% (*n* = 66/274) of all included tests. These included a range of molecular approaches, such as NAATs, RNA-based amplification assays, hybridization-based methods, and sequencing-based methods. Earlier studies primarily evaluated PCR-based NAATs and other amplification methods beginning in the 1990s, with a modest but steady annual output thereafter.

Conventional methods, such as mycobacterial culture and AFB smear microscopy on urine, date back to the 1990s. Notably, 76.0% (*n* = 19/25) of these tests were conducted in the context of UGTB diagnosis, and only four studies (six tests) evaluated their performance in diagnosing non-UGTB forms of TB.

Urine-based pathogen-derived assays predominated in the literature, accounting for more than 90% of all the studies. Urine-based host-derived biomarker assays were comparatively underrepresented, accounting for a small proportion of the research (21 studies).

### Urine-based antigen tests

The most studied LAM-based test was the LFA, with the Alere Determine LAM Ag being the most frequently evaluated (*n* = 72), followed by the Fujifilm SILVAMP TB LAM (*n* = 13). Among the 49 LAM-based tests other than LFAs, 43 studies investigated immunoassay-based methods, including ELISA and chemiluminescent enzyme immunoassay (CLEIA), for LAM detection. A few studies explored other types of LAM diagnostic approaches, including immuno-PCR and electrochemiluminescence (ECL) assays.

As shown in Figure 2 and the overall summary table (Supplementary material 3), there were markedly fewer publications on non-LAM urine antigen tests, including CFP (*n* = 10), Ag85B (*n* = 3), MPT64 (*n* = 2), and ESAT-6 (*n* = 2), than on LAM-based tests. Most cohorts included fewer than 100 participants (*n* = 15/19), whereas more than 75% of LAM studies included more than 100 participants. No studies reported the implementation of these assays in point-of-care (POC) formats, such as lateral flow devices, except for Turbawaty et al., who evaluated the TB antigen cocktail rapid immunochromatography test using ESAT-6, CFP-10, and MPT64 (Turbawaty et al., 2017; Turbawaty et al., 2023). The majority of non-LAM urine antigen tests were performed in a laboratory setting, predominantly using immunoassays.

Eight studies evaluated methods for urine concentration for antigen detection (Table 2). Most antigen-based studies incorporated urine concentration-targeted LAM, with input urine volumes ranging from 100 μL to 22 mL. Filtration-based concentration methods were most commonly employed, particularly centrifugal ultrafiltration devices such as Nanosep 10 K (Merck), Vivapore 10/20 mL (Sartorius Stedim), and Amicon filters (Millipore Sigma). A few studies reported diagnostic performance gains following concentration. For example, Savolainen et al. (2013) reported an increase in LAM detection sensitivity from 0.06 to 0.54 following Vivapore concentration. A 3 kDa molecular weight cut-off (MWCO) dialysis tubing (Spectra/Por) concentrated LAM from 100 μL urine, achieving high diagnostic accuracy (AUC, 0.95) in ELISA. Dass et al. (2023) employed a 100 kDa MWCO Amicon filter to concentrate MPT51 and MPT64 from 10 to 22 mL urine, yielding a sensitivity of 0.90 and specificity of 0.92 for microbiologically confirmed EPTB. Amin et al. (2022) applied a vacuum concentrator to 500 μL urine, achieving the highest reported LAM sensitivity of 0.98 and a specificity of 0.92 in PTB. While most studies used ELISA for antigen detection, Connelly et al. (2021) combined urine concentration with LFA-based LAM detection in the POC format.

**Table 2:** Summary of urine concentration methods.

Author (year)	TB type	Urine index test	Volume used	Concentration method	Diagnostic performance
Urine-based antigen detection					
Napolitano et al. (2008)	PTB	OCT (Rv1656), ELISA	NR	0.2-μm pore filters	Positivity: PTB (MRS+) 37.5% (*n* = 16), healthy 0% (*n* = 16)
Lawn et al. (2009)	PTB	LAM, ELISA (Chemogen)	NR	10 K Nanosep molecular filter (Merck)	PTB (MRS+, *n* = 58) Unconc: Sen 0.33, Conc: Sen 0.38
Savolainen et al. (2013)	PTB, EPTB	LAM, ELISA (Clearview)	12.5 mL	Vivapore 10/20 ml device (Sartorius Stedim)	PTB + EPTB (*n* = 35) vs. healthy (*n* = 101) Unconc: Sen 0.06, Spe 0.97, Conc: Sen 0.54, Spe 0.89
Amin et al. (2022)	PTB	LAM, Capture ELISA	500 μL	Vacuum concentrator (LabConco)	PTB (MRS+, *n* = 50) vs. non-TB (*n* = 50) Sen 0.98, Spe 0.92
Broger et al. (2020)	PTB	LAM, ECL	490 μL	Amicon Ultra-0.5 ml filter, 3 kDa cutoff (MilliporeSigma)	PTB (MRS+, *n* = 111) vs. non-TB (*n* = 261) Sen 0.67, Spe 0.98
Connelly et al. (2021)	PTB	LAM, LFA (DCN Diagnostics)	5 mL	Concentration Capture Reagent; Autovial 5 mL Syringeless Filter Device (Whatman)	PTB (MRS+, *n* = 126) vs. non-TB (*n* = 166) Sen 0.60, Spe 0.80
Amin et al. (2022)	ATB	LAM, Capture ELISA	100 μL	3 K MWCO dialysis tubing (Spectraphor)	PTB (CRS+, *n* = 41) vs. non-TB (*n* = 50) BJ76/A194-01 antibody: AUC 0.95
Dass et al. (2023)	EPTB	MPT51 and MPT64, ELISA	10–22 mL	100 kDa MWCO Amicon filter (MilliporeSigma)	Definite (*n* = 10)/probable (*n* = 77) vs. non-TB (*n* = 50) MPT51: definite: Sen 0.70, probable: Sen 0.33, Spe 0.86 MPT64: definite: Sen 0.90, probable: Sen 0.31, Spe 0.92
Urine-based NAATs					
Bordelon et al. (2017)	PTB	PCR	NR	Magnetic bead-based method	No significant difference in TB DNA levels between TB + (*n* = 33) vs. non-TB (*n* = 31), *p* = 0.132. Higher DNA levels in TB+/HIV + vs. TB-/HIV+, *p* = 0.037.
Patel et al. (2018)	PTB	PCR	NR	Filter column (10 μm pore size)	PTB (MRS+, *n* = 175) vs. non-TB (*n* = 237) Sen 0.43, Spe 0.89

ATB, active tuberculosis; AUC, area under the receiver operating characteristic curve; Conc, concentrated; CRS, composite reference standard; ECL, electrochemiluminescence; EPTB, extrapulmonary tuberculosis; kDa, kilodalton; LAM, lipoarabinomannan; LFA, lateral flow assay; MPT51, *Mycobacterium tuberculosis* Protein 51; MPT64, *Mycobacterium tuberculosis* Protein 64; MRS, microbiological reference standard; MWCO, molecular weight cut-off; NAATs, nucleic acid amplification tests; NR, not reported; OCT, ornithine carboamyltransferase; PCR, polymerase chain reaction; PTB, pulmonary tuberculosis; Sen, sensitivity; Spe, specificity; Unconc, unconcentrated.

### Urine-based NAATs

Among the 32 urine-based DNA NAATs (excluding the Xpert MTB/RIF assay) included in this review, 23 are summarized in Table 3: studies that exclusively targeted UGTB were excluded. The studies spanned 1995–2024 and included both PTB and EPTB cases with a range of reference standards. Overall, the technical methods used were highly variable, as described below.

**Table 3:** Summary of urine-based nucleic acid amplifications tests for *Mycobacterium tuberculosis.*

Author (year)	TB type	Index test	Storage	Collection	Preservatives	Centrifuge	DNA extraction	Target gene	Amplicon size, bp*	Diagnostic performance
Mitarai et al. (1995)	PTB	PCR	Stored, −20 °C	NR	NR	1700 g, 10 min, pellet	SDS, proteinase K; phenol-chloroform-isoamyl alcohol; ethanol	MPB64	240	Positivity: PTB (CRS+) 32.8% (*n* = 58), non-TB 12.5% (*n* = 51)
Gamboa et al. (1998)	EPTB	LCx assay	Stored, −30 °C	NR	NR	3,300 g, 20 min, pellet	LCx Respiratory Specimen Tube (Abbott)	Protein Antigen B	NR	EPTB (CRS+, *n* = 20) vs. non-TB (*n* = 49) Sen 0.70, Spe 1.00
Aceti et al. (1999)	PTB	Nested PCR	Stored, −80 °C	Morning, 3 days	NR	3,000 g, 20 min, pellet	NR	IS6110	566 (290)	Positivity: TB (MRS+) 100%, NTM 0%, healthy 0%
Kafwabulula et al. (2002)	PTB	Nested PCR	Stored, −70 °C	Spot	NR	4,000 g, 20 min, pellet	SDS, proteinase K; RNase; phenol-chloroform; isopropanol	IS6110	556 (123)	PTB (MRS+, *n* = 63) vs. Healthy (*n* = 63) Githui method: Sen 0.56, Spe 0.98 Sechi method: Sen 0.29, Spe 0.98
Marei et al. (2003)	ATB	PCR	Stored, 2–8°C	Morning, 3 days	NR	2000 g, 20 min, pellet	Lysozyme, SDS, proteinase K; phenol-chloroform; sodium acetate; ethanol	IS6110	317	TB (MRS+, *n* = 6) vs. Non-TB (*n* = 16) Sen 0.67, Spe 1.0
Torrea et al. (2005)	PTB EPTB	Nested PCR	Stored, −20 °C	Morning, 3 days	NR	3,000 g, 20 min, pellet	Heat lysis; freeze–thaw cycles; chloroform	IS6110	500 (300)	PTB (MRS+, *n* = 217)/EPTB (CRS+, *n* = 8) vs. healthy (*n* = 55) PTB: Sen 0.41, EPTB: Sen 0.57, Spe 0.98
Cannas et al. (2008)	PTB	Nested PCR	Stored, −80 °C	NR	EDTA, Tris–HCl	4,000 g, 20 min, pellet/supernatant	Guanidine isothiocyanate; silica-based Wizard Resin (Promega)	IS6110	129 (67)	Positivity: PTB (MRS+) 79.7% (*n* = 43), other 0% (*n* = 10), healthy 0% (*n* = 13)
Gopinath and Singh (2009)	PTB	PCR	Stored, −80 °C	Spot and morning, 3 days	NR	3,000 g, 20 min, pellet	Lysozyme, SDS, proteinase K; phenol-chloroform	hsp65 cfp32	441 (786)	Positivity: PTB (MRS+) 52.2% (*n* = 46), healthy 0% (*n* = 112)
da Cruz et al. (2011)	PTB EPTB	Nested PCR	Stored, −20 °C	Morning, 3 days	NR	4,500 rpm, 20 min, pellet	Heat lysis; silica-based resin (Sephagias); ethanol	IS6110	409 (316)	TB (CRS+, *n* = 58) vs. Non-TB + healthy (*n* = 98) Sen 0.40, Spe 0.91
Heydari et al. (2014)	PTB	PCR	NR	Morning	NR	NR	DNA extraction kit (Qiagen)	NR	NR	PTB (MRS+, *n* = 77) vs. healthy (*n* = 30) Sen 0.56, Spe 1.0
Lima et al. (2015)	PTB EPTB	Nested PCR	Stored, 4–8°C	3 days	NR	Centrifuge, pellet	Sephaglas BandPrep kit	IS6110	409 (316)	PTB (CRS+, *n* = 40)/EPTB (CRS+, *n* = 32) vs. Non-TB + healthy (*n* = 15) PTB: Sen 0.35, Spe 1.0, EPTB: Sen 0.41, Spe 1.0
Jamshidi Makiani et al. (2016)	PTB EPTB	Nested PCR	NR	Morning, 3 days	NR	3,000 g, 20 min	Heat lysis; freeze–thaw cycles; chloroform	16S rRNA	439 (123)	Positivity: TB (CRS+) 28% (*n* = 100)
Ayatollahi et al. (2016)	PTB	Nested PCR	Stored, 4 °C	NR	NR	4,500 rpm, 20 min, pellet	QIAmp DNA Mini Kit (Qiagen)	IS6110	209 (316)	PTB (MRS+, *n* = 60) vs. healthy (*n* = 30) Sen 0.30, Spe 1.0
Bordelon et al. (2017)	PTB	PCR	Stored, frozen	NR	NR	NR	Magnetic bead-based nucleic acid extraction	IS6110	67	TB DNA levels TB + (*n* = 33) vs. Non-TB (*n* = 31), *p* = 0.132. Higher DNA levels in TB+/HIV + vs. TB-/HIV+, *p* = 0.037.
Santos et al. (2018)	EPTB	qPCR	NR	Morning, 3 days	NR	Centrifuge, pellet	QIAmp DNA Mini Kit (Qiagen)	IS6110	122	EPTB (CRS+, *n* = 38) vs. Non-TB (*n* = 19) Sen 0.33, Spe 1.0
Patel et al. (2018)	PTB	PCR	Stored, −80 °C	Spot	EDTA	NR	Lysis/binding buffer, binding matrix, 10 μm pore size filter column	DR	38	PTB (MRS+, *n* = 175) vs. Non-TB (*n* = 237) Sen 0.43, Spe 0.89
Chemeda et al. (2019)	PTB	PCR	Stored, −80 °C	Morning	NR	3,000 g, 20 min, pellet	Lysozyme, SDS, proteinase K; CTAB/NaCl, phenol-chloroform-isoamyl alcohol; isopropanol	RD9	396	PTB (MRS+, *n* = 33) vs. MRS− (*n* = 84) Sen 0.73, Spe 0.89
Bisognin et al. (2020)	EPTB	PCR	NR	NR	NR	Centrifuge, pellet	MDR/MTB ELITe MGB1 Kit	IS6110	NR	EPTB (MRS + *n* = 20) vs. MRS− (*n* = 20) Sen 0.80, Spe 1.0
Costa-Lima et al. (2020)	PTB EPTB	Nested PCR	NR	3 days	NR	Centrifuge, pellet	QIAmp DNA Mini Kit (Qiagen)	IS6110	409 (316)	TB (CRS+, *n* = 39) vs. Non-TB (*n* = 43) Sen 0.28, Spe 0.98
Oreskovic et al. (2021)	PTB	PCR	Stored, −80 °C	Spot and morning	EDTA, Tris–HCl	8,000 g, 5 min, supernatant	Magnetic bead-based hybridization capture	IS6110	40	PTB (MRS+, *n* = 49) vs. Non-TB (*n* = 24) Sen 0.84, Spe 1.0
Araújo et al. (2023)	EPTB	IS6110-LAMP	NR	Morning	NR	Centrifuge, pellet	QIAamp DNA Mini Kit (Qiagen)	IS6110	NR	EPTB (CRS+, *n* = 24) vs. Non-TB (*n* = 24) Sen 0.79, Spe 0.71
Tschan et al. (2024)	PTB	qPCR	Stored, −80 °C	NR	EDTA, Tris–HCl	4,347 g, 10 min, supernatant	Magnetic bead-based hybridization capture	IS6110	NR	PTB (MRS+, *n* = 53) vs. Contacts (*n* = 59) Sen 0.72, Spe 0.96
Salazar et al. (2024)	PTB EPTB	Nested PCR	NR	Morning, 3 days	NR	Centrifuge, pellet	Qiagen Mini Prep Kit (Qiagen)	IS6110	409 (316)	PTB (CRS+, *n* = 77) vs. Non-TB (*n* = 38) Sen 0.61, Spe 0.52

16S rRNA, 16S ribosomal ribonucleic acid; cfp32, 32-kilodalton culture filtrate protein; CRS, composite reference standard; DNA, deoxyribonucleic acid; DR, direct repeat; EDTA, ethylenediaminetetraacetic acid; EPTB, extrapulmonary tuberculosis; hsp65, 65-kilodalton heat shock protein; IS6110, insertion sequence 6,110; LAMP, loop-mediated isothermal amplification; LCx assay, ligase chain reaction assay; MPB64, mycobacterium protein b 64; MRS, microbiological reference standard; NR, not reported; NTM, nontuberculous mycobacteria; PCR, polymerase chain reaction; PTB, pulmonary tuberculosis; qPCR, quantitative polymerase chain reaction; RD9, region of difference 9; SDS, sodium dodecyl sulfate; Sen, sensitivity; Spe, specificity; TB, tuberculosis; Tris–HCl, tris (hydroxymethyl) aminomethane hydrochloride.

Urine collection protocols varied, with some studies specifying early-morning specimens (*n* = 12/23) or collecting specimens over three consecutive days (*n* = 10/23) to maximize analyte concentration. All assays were performed on stored urine samples, with most samples frozen prior to analysis. In more recent studies, preservatives such as EDTA and/or Tris–HCl were occasionally added to prevent nuclease-mediated DNA degradation (*n* = 4/23). Centrifugation was commonly employed either for decontamination or to remove cellular debris. While earlier studies predominantly analyzed the pellet fraction (*n* = 17/23), more recent investigations used the supernatant (*n* = 3).

DNA extraction methods also varied widely, from classical chemical extraction (e.g., SDS/proteinase K followed by phenol-chloroform and alcohol precipitation) to commercial kits, magnetic bead-based capture, and silica-based resins. Several studies employed mechanical lysis methods, such as freeze–thaw cycles or heat lysis, in combination with organic extraction. While recent studies commonly utilized commercial kits such as the QIAmp DNA Mini Kit (QIAGEN), earlier studies employed diverse extraction methodologies.

The most frequently targeted genes were IS6110 (*n* = 16/23), followed by MPB64, protein antigen B, hsp65, cfp32, 16S rRNA, direct repeat (DR), and region of difference 9 (RD9). Amplicon sizes ranged from 38 bp to >300 bp. For nested PCR, the inner amplicons ranged from 67 to 316 bp. None of the included studies employed a multiplex approach.

Two studies explicitly evaluated urine concentrations before urine-based NAATs. Bordelon et al. (2017) used a magnetic bead-based DNA capture system and found no significant difference in DNA levels between participants with and without TB (*p* = 0.132); however, higher DNA levels were detected in individuals with TB+/HIV + than in those with TB–/HIV + (*p* = 0.037). Patel et al. (2018) employed a filter column with a 10 μm pore size, reporting a sensitivity of 0.43 and a specificity of 0.89 in PTB. Most other NAATs used centrifugation as part of routine decontamination or debris removal, but did not specify this as a deliberate concentration step; therefore, these were not included in Table 2.

### Urine-based host-derived biomarker assays

In contrast to pathogen-derived assays, host-derived biomarker assays were underrepresented, accounting for a limited number of studies (10%, *n* = 21/208) and emerging primarily in recent years. These studies employed platforms such as ELISA, multiplex immunoassays, nucleic magnetic resonance, and mass spectrometry to identify diverse biological signatures. Proteomic investigations frequently evaluated immune mediators, such as interferon-*γ*-inducible protein 10 and neopterin, which was consistently elevated in patients with PTB compared to healthy controls (Elvira, 2021; Petrone et al., 2015; Yuksekol et al., 2003). Combinations of several biosignatures were evaluated to aim high sensitivity (Eribo et al., 2020; Wang et al., 2018). Metabolomic and lipidomic profiling identified distinct fingerprints characterized by dysregulation in amino acids and energy metabolism (Izquierdo-Garcia et al., 2020; Fitzgerald et al., 2019). Additionally, colorimetric sensor arrays detected host-derived volatile organic compound signatures from urine, which was capable to distinguishing TB cases, although diagnostic performance was notably lower in HIV-positive individuals (Lim et al., 2016; Sandlund et al., 2018).

## Discussion

This scoping review mapped nearly three decades of research on urine-based diagnostic tests for TB and highlighted key trends, gaps, and technical challenges across antigen- and NAAT-based approaches. Overall, the literature trend indicates a growing interest in urine as a non-invasive diagnostic specimen, while also revealing persistent limitations in population coverage, disease spectrum, and underdeveloped assays. From a global health perspective, urine-based diagnostics have particular relevance for decentralized TB care in high-burden, resource-limited settings. Non-sputum-based tests could facilitate earlier diagnosis among populations that are currently underserved by sputum-dependent algorithms (e.g., children, people with EPTB, severely ill patients). However, the predominance of laboratory-based assays and the limited evaluation of POC formats beyond urine LAM highlight a translational gap between diagnostic innovation and real-world implementation.

### Overview of research trend

Publications increased steadily from the early 2000s, with 71.2% published in the past decade. This growth reflects the increasing recognition of urine as a practical, non-invasive specimen, particularly in high-burden settings where sputum collection is often challenging, and non-sputum alternatives are urgently needed. Across assay categories, antigen-based diagnostics dominate the literature, largely driven by the emergence of LAM-based tests.

Despite this expansion, crucial gaps remain in the literature. PTB has been more frequently studied than EPTB, despite the diagnostic challenge posed by EPTB and the potential value of urine as a non-site-specific specimen. Likewise, most studies enrolled adults, with relatively few focusing on children, a population in which sputum-based diagnosis is particularly difficult. Although LAM-based tests are the most extensively studied urine diagnostic methods, their sensitivity is suboptimal for non-HIV individuals. Nevertheless, studies that specifically evaluate urine-based diagnostic tests in non-HIV-infected individuals remain limited. These findings highlight a mismatch between the populations most in need of broadly applicable, non-invasive TB diagnostics and those most frequently represented in the literature.

### Urine-based antigen tests

LAM-based tests, particularly LFAs, such as Alere Determine LAM Ag and Fujifilm SILVAMP TB LAM, were the most frequently cited in the literature, accounting for 60% of the included studies. Their predominance reflects the ease of POC use, commercial availability, and strong evidence of HIV-associated TB (Broger et al., 2023; Nathavitharana et al., 2021; Seid et al., 2022; Sood et al., 2023; Gupta-Wright et al., 2016; Bjerrum et al., 2019; Shah et al., 2016), although their sensitivity remains limited in non-HIV individuals (Minion et al., 2011).

In contrast, research on non-LAM urine antigens remains sparse and largely limited to small-scale studies using laboratory-based assays such as ELISA. The near absence of POC implementations (Turbawaty et al., 2017; Turbawaty et al., 2023) highlights important translational barriers. Collectively, these findings indicate that non-LAM urine antigens currently remain proof-of-concept evaluations and that substantial methodological refinement and validation will be required before they can be considered viable alternative or complements to LAM-based diagnostics.

Methods for urine concentration have been explored primarily for antigen detection, particularly for LAM. Across studies, improved diagnostic performance following concentration suggest that pre-analytical processing is a key determinant of assay yield. The variability in reported performance highlights that factors such as concentration mechanism, MWCO, and input urine volume critically influence antigen recovery (Lawn et al., 2009; Savolainen et al., 2013; Dass et al., 2023; Amin et al., 2022; Broger et al., 2020; Amin et al., 2018).

The limited number of published studies on non-LAM urine antigens may partly reflect a publication bias (DeVito & Goldacre, 2019; Schmucker et al., 2014), whereby assays with negative or inconclusive findings are less likely to be reported, raising the possibility that some candidate antigens may have limited diagnostic utility. Nonetheless, the findings of this review clearly indicate that the systematic exploration of non-LAM urine antigens has been minimal, rather than conclusively demonstrating a lack of usefulness. This gap is particularly important, given the known limitations of LAM-based diagnostics, including suboptimal sensitivity in non-HIV individuals and concerns regarding cross-reactivity with non-tuberculous mycobacteria. Collectively, these considerations suggest that further research on MTB-specific antigens is warranted to complement or enhance existing LAM-based diagnostics.

### Urine-based NAATs

Urine-based NAATs constitute a substantial component of urine-based diagnostic literature. Across the included studies, urine-based NAATs were evaluated in a range of clinical contexts, with earlier work focusing predominantly on UGTB, and more recent studies increasingly targeting PTB and other forms of EPTB. This shift reflects growing interest in using urine as a non-sputum, non-site-specific specimen for TB diagnosis beyond the genitourinary tract. Urine-based nucleic acid detection differs fundamentally between UGTB and non-UGTB disease. In UGTB genomic DNA from intact MTB bacilli shed into urine can be detected using conventional NAAT workflow. In contrast, non-UGTB disease requires detection of fragmented cell-free trDNA, necessitating ultrashort amplicon design and recovery of soluble DNA. Because these methodological requirements directly influence assay sensitivity and interpretation, studies explicitly focused on UGTB were excluded from target urine-based NAAT synthesis. Accumulating evidence focused on methodological optimization of trDNA detection will be critical for improving diagnostic sensitivity.

Most DNA-based urine diagnostics aim to detect trDNA, which comprises short cell-free DNA fragments released from dying human cells and microorganisms into the circulation, filtered through the kidneys, and excreted in urine (Green et al., 2009; Oreskovic et al., 2021; Patel et al., 2018; Cannas et al., 2008). Cannas et al. (2008) provided early biological evidence of the transrenal origin of MTB DNA, showing that short amplicons (67 bp) were mainly detectable in the urine supernatant, whereas longer targets (>300 bp) were not. These findings support the hypothesis that small cell-free MTB DNA fragments from distant infection sites can be detected in urine.

Across studies, urine pretreatment varied considerably reflecting evolving assumptions about the biological nature of trDNA. Early studies extracted DNA from urine sediments (pellets), likely reflecting assumptions that intact MTB bacilli were present (Mitarai et al., 1995; Sechi et al., 1997; Aceti et al., 1999; Kafwabulula et al., 2002), whereas later studies showed that trDNA is largely soluble and concentrated in the supernatant (Green et al., 2009; Cannas et al., 2008). This biological insight has important methodological implications, as workflows that discard the supernatant may inadvertently reduce analytical sensitivity. The limited number of studies explicitly evaluating concentration strategies for trDNA detection underscores the need for systematic investigation of pretreatment approaches optimized for trDNA recovery.

Various methods have been developed to improve the trDNA recovery. This reflects ongoing efforts to optimize recovery of trDNA from urine. The adoption of chaotropic agents, solid-phase capture systems and sequence-specific enrichment strategies highlights the recognition that conventional extraction methods may be suboptimal for trDNA (Oreskovic et al., 2021; Bordelon et al., 2017; Patel et al., 2018; Cannas et al., 2008; Tschan et al., 2024). The widespread use of commercial extraction kits in more recent studies likely reflects their reproducibility and convenience. However, their performance for trDNA has not been systematically evaluated.

The predominance of IS6110 as a diagnostic target likely reflects its multicopy nature within the MTB genome, which enhances detection when the DNA concentration is low (Oreskovic et al., 2021; Marangu et al., 2015). Other multicopy regions, including the DR region, were also used (Green et al., 2009; Patel et al., 2018). Increasing use of ultrashort amplicons in more recent studies is biologically consistent with the fragmented nature of trDNA (Oreskovic et al., 2021; Bordelon et al., 2017; Patel et al., 2018). These trends highlight the importance of target selection and amplicon design as key determinants of assay performance in urine-based NAATs.

Overall, urine-based NAATs exhibited pronounced heterogeneity in technical approaches, particularly with respect to urine pretreatment, DNA extraction methods, and primer and amplicon design. This lack of standardization complicates the direct comparison of diagnostic accuracy and likely contributes to the wide variation in reported sensitivities. These variations reflect the early stages of assay development and highlight the need for further investigation to validate urine-based NAATs for TB diagnosis.

Evidence for urine concentration strategies using NAATs is limited. The heterogeneous findings observed across the few studies evaluated concentration methods suggests that performance is strongly influenced by host factors, with potentially greater utility in immunocompromised populations (Bordelon et al., 2017). Although not included in our extraction table, Lawn et al. (2017) and Kerkhoff et al. (2017) reported that large-volume centrifugation (30–40 mL) prior to Xpert MTB/RIF improved detection from 42.2 to 59.0% for PTB and EPTB and from 53.7 to 78.0% for disseminated TB. These improvements are likely driven by the recovery of intact bacilli or larger DNA fragments. This contrasts with trDNA-focused assays, which primarily require supernatant recovery (Green et al., 2009), emphasizing that concentration strategies must be tailored to the biological target: pellets for bacilli or large DNA, supernatants for trDNA, or combined approaches for broader capture.

### Urine-based host-derived biomarker assays

Studies on urine-based host-derived biomarker assays for TB remains limited and exploratory. Most studies adapted candidate biomarkers originally identified in blood-based host-response signatures rather than biomarkers specifically discovered and optimized for urine (Elvira, 2021). Biologically, these biomarkers reflect systemic immune and metabolic perturbations induced by TB, interferon-*γ*-mediated inflammation and altered energy metabolism (Elvira, 2021; Yuksekol et al., 2003; Izquierdo-Garcia et al., 2020). However, such host responses lack disease specificity and may overlap with other infectious or inflammatory conditions common in TB-endemic settings (Petrone et al., 2015; Yuksekol et al., 2003). HIV co-infection further modifies host signatures, complicating interpretation (Eribo et al., 2020; Sandlund et al., 2018). The limited number of studies shown in the scoping review suggests that urine-based host-derived biomarker research remains in an early phase. Despite current limitations in study size and heterogeneity, these approaches may serve as promising adjunctive tools. Further studies with larger and diverse cohorts are needed to establish their diagnostic utility.

### Strengths and limitations

A key strength of this scoping review is the comprehensive mapping of nearly three decades of urine-based diagnostic studies for TB, encompassing both antigen- and NAAT-based approaches. Although we deliberately precluded quantitative synthesis or meta-analysis owing to marked heterogeneity, this allowed us to focus on critical technical approaches beyond diagnostic performance. Specifically, this review highlights how variables such as urine pretreatment procedures and concentration methods can substantially influence assay yield but remain poorly addressed in conventional reviews. By systematically capturing these dimensions, this review provides practical insights into the validation of reliable TB diagnostics.

Additionally, the limitations of this study must be acknowledged. We excluded very small cohorts and purely analytical studies based on spiked-in experiments, which may have led us to overlook some early-stage diagnostic innovations. Owing to the rapid evolution of the field, emerging technologies such as CRISPR-based assays, next-generation sequencing, and advanced biosensors remain underrepresented. Some included studies enrolled mixed EPTB cohorts that also contained patients with UGTB without stratifying reporting, making complete separation at the study level infeasible. In addition, subclinical or unrecognized genitourinary involvement cannot be entirely excluded in patients classified as non-UGTB, particularly disseminated disease. To minimize selection bias, such studies were retained for descriptive mapping but excluded from targeted urine-based NAAT analyses. Publication bias is a major limitation of the available evidence (DeVito & Goldacre, 2019; Schmucker et al., 2014). Studies reporting negative or inconclusive findings, particularly those evaluating novel antigens, DNA targets, or pre-analytical methods, are less likely to be published, resulting in the selective visibility of assays with positive or promising performance. This bias is likely to be pronounced in urine-based TB diagnostics, where biological signals are often weak, and assay optimization is challenging. Consequently, the current literature may overrepresent successful approaches while underrepresenting failed or suboptimal strategies, thereby limiting our ability to draw balanced conclusions or identify unproductive research directions.

## Conclusion

In conclusion, LAM has been extensively investigated as a urine-based diagnostic test for TB. Beyond LAM, research on non-LAM urine antigens and urine-based NAATs has expanded over the past decade, demonstrating proof-of-concept diagnostic potential; however, these remain constrained by the small number of studies, small cohort sizes, and predominance of laboratory-based evaluations. The diversity of technical approaches observed across studies on non-LAM urine tests reflects an exploratory research landscape, highlighting that no robust, broadly applicable urine-based diagnostic tests beyond LAM have yet been established. While urine concentration methods may enhance assay yield, the lack of systematic, head-to-head comparisons between concentrated and unconcentrated urine limits firm conclusions regarding the incremental value of concentration methods. Overall, further research is required in these areas (i.e., non-LAM urine antigen, urine-based NAATs, and urine concentration methods) to identify and validate reliable urine-based diagnostic tests for TB. Urine-based diagnostics represent a promising yet underexploited approach to TB diagnosis. Together, these findings highlight the importance of aligning future urine-based diagnostic development with WHO target product profiles and implementation priorities in high-burden settings.

## Data Availability

The original contributions presented in the study are included in the article/Supplementary material, further inquiries can be directed to the corresponding author.

## Footnotes

1 https://osf.io/u7r92/.
