# Supplementary material for: Urine-based diagnostic tests for tuberculosis: a scoping review highlighting unmet diagnostic needs
Source: Front Microbiol. 2026 Mar 27;17:1783312. doi: 10.3389/fmicb.2026.1783312 (PMC13066143; doi:10.3389/fmicb.2026.1783312)
Supplement: Supplementary file 2 [file data_sheet_2.pdf]

Supplementary 2. Search strategy

Key words for database search: urine, diagnosis, tuberculosis

Table. Search strategy

Database	Search strategy
MEDLINE (PubMed)	(urin*[tw]) AND (diagnosis[MESH] OR diagnos*[tw] OR detect*[tw] OR identif*[tw]) AND (TB[ti] OR tuberculosis[ti]) NOT (animals[MESH] NOT humans[MESH])
Scopus	(TITLE-ABS-KEY(urin*)) AND (INDEXTERMS(diagnosis) OR TITLE-ABS-KEY(diagnos*) OR TITLE-ABS-KEY(detect*) OR TITLE-ABS-KEY(identif*)) AND (TITLE(TB) OR TITLE(tuberculosis)) AND NOT (INDEXTERMS(animals) NOT INDEXTERMS(humans))
Embase (Ovid)	(urin*.mp.) AND (exp diagnosis/ OR diagnos*.mp. OR detect*.mp. OR identif*.mp.) AND (TB.ti. OR tuberculosis.ti.)

For Embase, "English" and "Human" filters were used.
