# Supplementary material for: Urine-based diagnostic tests for tuberculosis: a scoping review highlighting unmet diagnostic needs
Source: Front Microbiol. 2026 Mar 27;17:1783312. doi: 10.3389/fmicb.2026.1783312 (PMC13066143; doi:10.3389/fmicb.2026.1783312)
Supplement: Supplementary file 3 [file data_sheet_3.pdf]

Supplementary 3. Summary of urine-based diagnostic tests for tuberculosis

Author Year	Age	TB type	Patient setting	Country	HIV status	Urine index test	Sample size	Reference standard	Diagnostic performance	STARD
**Urine LAM lateral flow assays (79 publications, 86 tests)**										
Peter JG 2012 (1)	Adult	PTB EPTB	In	South Africa	PLWH	Alere LAM	241 214	1. MRS (culture) 2. CRS	1. Sen 0.66, Spe 0.66 2. Sen 0.60, Spe 0.96	Yes
Lawn SD 2012 (2)	Adult	PTB	Out	South Africa	PLWH	Alere LAM	516	MRS (culture)	Sen 0.28, Spe 0.99	Yes
Lawn SD 2012 (3)	Adult	PTB	Out	South Africa	PLWH	Alere LAM	84	MRS (culture)	Sen 0.29	NR
Lawn SD 2013 (4)	Adult	PTB	Out	South Africa	PLWH	Alere LAM	81	MRS (culture)	Sen 0.27	NR
Peter JG 2013 (5)	Adult	PTB EPTB	In	South Africa	PLWH	Alere LAM	214	CRS	Sen 0.46, Spe 0.96	NR
Nakiyingi L 2014 (6)	Adult	PTB	Out & In	Uganda, South Africa	PLWH	Alere LAM	940	MRS (culture)	Sen 0.37, Spe 0.98	Yes
Balcha TT 2014 (7)	Adult	PTB EPTB	NR	Ethiopia	PLWH	Alere LAM	757	MRS (culture, Xpert)	Sen 0.26, Spe 0.93	NR
Nicol MP 2014 (8)	Children	PTB	NR	South Africa	PLWH Non-HIV	Alere LAM	535	MRS (culture)	Sen 0.48, Spe 0.61	NR
Drain PK 2014 (9)	Adult	PTB	Out	South Africa	PLWH	Alere LAM	342	1. MRS 2. CRS	1. Sen 0.28, Spe 0.90 2. Sen 0.25, Spe 0.91	Yes
Kerkhoff AD 2014 (10)	Adult	PTB	Out	South Africa	PLWH	Alere LAM	485	MRS (culture)	Sen 0.28, Spe 0.99	NR
Manabe YC 2014 (11)	Adult	ATB	In	Uganda	PLWH	Alere LAM	330	MRS (culture)	Sen 0.62, Spe 0.81	NR
Bjerrum S 2015 (12)	Adult	PTB	Out & In	Ghana	PLWH	Alere LAM	469	1. MRS (culture, Xpert) 2. CRS	1. Sen 0.44, Spe 0.95 2. Sen 0.36, Spe 0.98	Yes
Kroidl I 2015 (13)	Children	PTB	Out	Tanzania	PLWH Non-HIV	Alere LAM	53 89	1. MRS (culture, Xpert) 2. CRS	1. Sen 0.28, Spe 0.97 2. Sen 0.13, Spe 0.97	NR
Nakiyingi L 2015 (14)	Adult	ATB	Out & In	Uganda	PLWH	Alere LAM	417	MRS (culture)	Sen 0.38, Spe 0.93	NR
Peter JG 2015 (15)	Adult	PTB	Out	South Africa, Zimbabwe, Zambia, Tanzania	PLWH	Alere LAM	569	MRS (culture, Xpert, AFB)	Sen 0.23, Spe 0.93	Yes
Drain PK 2015 (16)	Adult	PTB	Out	South Africa	PLWH	Alere LAM	320	1. MRS (cutlure) 2. CRS	1. Sen 0.41, Spe 0.92 2. Sen 0.38, Spe 0.93	NR
Cox JA 2015 (17)	Adult	UGTB	NA (Autopsy)	Uganda	PLWH	Alere LAM	36	CRS	Sen 0.81, Spe 1.0	NR
Hanifa Y 2016 (18)	Adult	PTB EPTB	Out	South Africa	PLWH	Alere LAM	424	CRS	Sen 0.14, Spe 0.96	NR
Drain PK 2016 (19)	Adult	PTB	Out	South Africa	PLWH	Alere LAM	675	MRS (culture)	Sen 0.31, Spe 0.92	NR
Drain PK 2016 (20)	Adult	PTB	Out	South Africa	PLWH Non-HIV	Alere LAM	90	MRS (culture)	Sen 0.42, Spe 0.85	Yes
Zijenah LS 2016 (21)	NR	PTB	In	Zimbabwe	PLWH	Alere LAM	457	1. MRS (culture) 2. CRS	1. Sen 0.61, Spe 0.86 2. Sen 0.49, Spe 0.98	NR
Peter JG 2016 (22)	Adult	ATB	In	South Africa, Tanzania, Zambia, Zimbabwe	PLWH	Alere LAM	1172	MRS (AFB, Xpert, culture)	Sen 0.46, Spe 0.89	NR

Suwanpimolkul G<br>2017 (23)	NR	PTB<br>DTB	NR	Thailand	PLWH<br>Non-HIV	Alere LAM	63	CRS	Sen 0.37, Spe 0.85	NR
Lawn SD<br>2017 (24)	Adult	PTB<br>EPTB	In	South Africa	PLWH	Alere LAM	413	MRS (Xpert, culture)	Sen 0.39, Spe 0.99	Yes
Gina P<br>2017 (25)	Adult	PTB	In	South Africa	PLWH	Alere LAM	41<br>123	1. MRS (culture, Xpert)<br>2. CRS	Spot vs. Early morning<br>1. Sen 0.12 vs. 0.39<br>2. Sen 0.1 vs. 0.3	Yes
Sahle SN<br>2017 (26)	Adult	PTB	NR	Ethiopia	PLWH<br>Non-HIV	Alere LAM	122	MRS (culture)	Sen 0.37, Spe 0.98	NR
Florida M<br>2017 (27)	Adult	ATB	NR	Mozambique	PLWH	Alere LAM	972	MRS (Xpert)	Positivity: MRS+ 32.4%,<br>MRS- 1.1%	NR
Huerga H<br>2017 (28)	Adult	PTB	Out & In	Kenya	PLWH	Alere LAM	474	MRS (culture, Xpert)	Sen 0.65, Spe 0.84	NR
Kerkhoff AD<br>2017 (29)	Adult	DTB	In	South Africa	PLWH	Alere LAM	132	MRS (culture)	Positivity: MRS+ 78%, MRS-<br>33%	NR
Boyles TH<br>2018 (30)	Adult	PTB	In	South Africa	PLWH	Alere LAM	332	MRS (culture)	Sen 0.36, Spe 0.93	NR
LaCourse SM<br>2018 (31)	Children	ATB	In	Kenya	PLWH	Alere LAM	129	MRS (culture, Xpert)	Sen 0.43, Spe 0.91	NR
Gautam H<br>2019 (32)	Children	ITTB<br>LNTB	Out	India	NR	Alere LAM	280<br>101	ITTB: MRS<br>LNTB: MRS	ITTB: Sen 0.73, Spe 0.73<br>LNTB: Sen 0.76, Spe 0.70	NR
Broger T<br>2019 (33)	Adult	PTB<br>EPTB	In	South Africa	PLWH	Fuji LAM<br>Alere LAM	968	1. MRS (culture, Xpert),<br>2. CRS	Fuji LAM:<br>1. Sen 0.70, Spe 0.91<br>2. Sen 0.65, Spe 0.96<br>Alere LAM:<br>1. Sen 0.42, Spe 0.95<br>2. Sen 0.38, Spe 0.98	Yes
Van Hoving DJ<br>2019 (34)	Adult	PTB<br>EPTB	Out<br>(ED)	South Africa	PLWH	Alere LAM	411	MRS (culture, Xpert)	Sen 0.42, Spe 0.91	NR
Younis H<br>2019 (35)	Adult	PTB	NR	South Africa	PLWH	Alere LAM	104	MRS (Xpert, AFB, culture)	Sen 0.3, Spe 1.0	Yes
Songkhla MN<br>2019 (36)	Adult	PTB<br>EPTB	Out	Thailand	PLWH	Alere LAM	280	1. MRS (culture, PCR)<br>2. CRS	1. Sen 0.75, Spe 0.76<br>2. Sen 0.61, Spe 0.86	NR
Yoon C<br>2019 (37)	Adult	PTB	Out	Uganda	PLWH	Alere LAM	439	MRS (culture)	Sen 0.26, Spe 0.98	Yes
Byashalira K<br>2019 (38)	Adult	PTB	Out & In	Tanzania	PLWH	Alere LAM	84	MRS (Xpert)	Positivitiy: MRS+ 50%,<br>MRS- 25.9%	NR
Esmail A<br>2020 (39)	Adult	PTB	In	South Africa,<br>Tanzania,<br>Zambia,<br>Zimbabwe	PLWH	Alere LAM	561	MRS (culture)	Sen 0.38, Spe 0.88	NR
Andama A<br>2020 (40)	Adult	PTB	Out & In	Uganda	PLWH<br>Non-HIV	Alere LAM	357	MRS (culture, Xpert)	Sen 0.08, Spe 0.99	Yes
Kerkhoff AD<br>2020 (41)	Adult	PTB<br>EPTB	In	South Africa	PLWH	Fuji LAM<br>Alere LAM	553	MRS (culture, Xpert)	Fuji LAM: Sen 0.60–0.91<br>Alere LAM: Sen 0.19–0.61	NR
Pasipamire M<br>2020 (42)	Adult	PTB	Out<br>(Ante/pos<br>tnatal)	Eswatini	PLWH	Alere LAM	372	MRS (culture)	Sen 0.11, Spe 0.94	NR
Tlali M<br>2020 (43)	Adult	PTB	Out	South Africa	PLWH	Alere LAM	105	MRS (culture)	Sen 0.42	NR
Broger T<br>2020 (44)	Adult	PTB	Out	South Africa,<br>Peru	Non-HIV	Alere LAM<br>Fuji LAM	372	MRS (culture, Xpert)	Alere LAM: Sen 0.11, Spe<br>0.92	Yes

								Fuji LAM: Sen 0.53, Spe 0.99	
Bjerrum S 2020 (45)	Adult	ATB	Out & In	Ghana	PLWH	Alere LAM<br>Fuji LAM	450	MRS (culture, Xpert)	Alere LAM:<br>Sen 0.53, Spe 0.96<br>Fuji LAM:<br>Sen 0.74, Spe 0.89	Yes
Garcia JI 2020 (46)	NR	PTB<br>EPTB	Out & In	Guatemala	PLWH	Alere LAM	291	MRS (culture, Xpert)	Sen 0.57, Spe 0.90	NR
Gupta A 2020 (47)	NR	PTB	In	India	Non-HIV	Alere LAM	56	MRS (Xpert)	Sen 0.48, Spe 0.83	NR
Grant AD 2020 (48)	Adult	ATB	Out	South Africa	PLWH	Alere LAM	968	MRS (culture)	Positivity: MRS+ 42%, MRS- 12%	NR
Cresswell FV 2020 (49)	Adult	EPTB (TBM)	In	Uganda	PLWH	Alere LAM	40<br>22	CRS (definite, probable)	Positivity: Definite 36.8%, Probable 40.9%	NR
Huerga H 2020 (50)	Adult	PTB	Out	Malawi	PLWH	Alere LAM	312	MRS (Xpert)	Positivity: MRS+ 52.3%, MRS- 13.1%	NR
van Hoving DJ 2020 (51)	Adult	PTB<br>EPTB	Out (ED)	South Africa	PLWH	Alere LAM	414	MRS (culture, Xpert)	Positivity: MRS+ 41.8%, MRS- 9.5%	NR
Comella-Del-Barrio P 2021 (52)	Adult	PTB	Out	Nigeria	PLWH<br>Non-HIV	Fuji LAM	204	MRS (culture, Xpert)	Sen 0.67, Spe 0.96	NR
Comella-Del-Barrio P 2021 (53)	Children	PTB<br>EPTB	NR	Haiti	NR	Fuji LAM	79	1. MRS (Xpert)<br>2.CRS	1. Sen 0.60, Spe 0.95<br>2. Sen 0.11, Spe 0.92	NR
Nicol MP 2021 (54)	Children	PTB	NR	South Africa	PLWH<br>Non-HIV	Fuji LAM<br>Alere LAM	204	MRS (culture, Xpert)	Fuji LAM:<br>Sen 0.42, Spe 0.92<br>Alere LAM:<br>Sen 0.50, Spe 0.66	NR
Connelly JT 2021 (55)	Adult	PTB	Out & In	Uganda	PLWH<br>Non-HIV	LAM LFA	292	MRS (culture, Xpert)	Sen 0.60, Spe 0.80	Yes
de Vasconcellos K 2021 (56)	Adult	ATB	In<br>(ICU)	South Africa	PLWH<br>Non-HIV	Alere LAM	50	1. MRS (culture, Xpert)<br>2. CRS	1. Sen 0.50, Spe 0.84<br>2. Sen 0.48, Spe 0.93	Yes
Nkereuwem E 2021 (57)	Children	PTB	Out	Gambia, Mali, Nigeria, Tanzania	PLWH<br>Non-HIV	Alere LAM<br>Fuji LAM	415	1. MRS (culture, Xpert)<br>2. CRS	Alere LAM:<br>1. Sen 0.31, Spe 0.88<br>2. Sen 0.20, Spe 0.90<br>Fuji LAM:<br>1. Sen 0.65, Spe 0.84<br>2. Sen 0.33, Spe 0.83	Yes
Muyoyeta M 2021 (58)	Adult	PTB	NR	Zambia	PLWH<br>Non-HIV	Fuji LAM	151	MRS (culture)	Sen 0.77, Spe 0.92	NR
Kebede W 2021 (59)	Adult	PTB	In	Ethiopia	PLWH	Alere LAM	52	MRS (culture)	Positivity: MRS+ 60%, MRS- 0%	NR
Schramm B 2021 (60)	Children	ATB	In	Niger	PLWH<br>Non-HIV	Alere LAM	122	CRS	Positivity: CRS+ 22.7%, CRS- 1%	NR
Chernick L 2021 (61)	Adult	PTB<br>EPTB	In	South Africa	PLWH	Alere LAM	48	MRS (culture, Xpert)	Positivity: MRS+ 71%	NR
Simieneh A 2022 (62)	NR	EPTB	NR	Ethiopia	PLWH<br>Non-HIV	Alere LAM	126	1. MRS (culture)<br>2. CRS	1. Sen 0.35, Spe 0.91<br>2. Sen 0.33, Spe 0.94	NR
Boloko L 2022 (63)	NR	ATB	In	South Africa	PLWH	Alere LAM	375	MRS (Xpert)	Sen 0.46	Yes
Orikiriza P 2022 (64)	Children	PTB<br>EPTB	NR	Uganda	PLWH<br>Non-HIV	Alere LAM	209<br>195	1. MRS (culture, Xpert)<br>2. CRS	1. Sen 0.50, Spe 0.75<br>2. Sen 0.25, Spe 0.73	NR

Shapiro AE 2022 (65)	Adult	PTB	Out & In	South Africa	PLWH Non-HIV	Alere LAM	130	MRS (culture, Xpert)	Sen 0.35, Spe 1.0	NR
Kanyama C 2022 (66)	Adult	PTB	In	Malawi	PLWH	LAM LFA	363	Urine Xpert positive	Sen 0.81, Spe 0.80	NR
Indirawati NN 2022 (67)	Adult	EPTB	NR	Indonesia	PLWH	Fuji LAM	62	1. MRS (culture, Xpert)<br>2. CRS	1. Sen 0.75, Spe 0.74<br>2. Sen 0.61, Spe 0.92	NR
Bjerrum S 2022 (68)	Adult	ATB	Out & In	Ghana	PLWH	Fuji LAM	389	1. MRS (culture, Xpert)<br>2. CRS	Spot vs. Early morning<br>1. Sen 0.67 vs 0.70, Spe 0.90 vs 0.89<br>2. Sen 0.60 vs 0.60, Spe 0.92 vs 0.91	NR
Wake RM 2022 (69)	Adult	PTB EPTB	Out & In	South Africa	PLWH	Alere LAM	100	MRS (culture)	Sen 0.71, Spe 0.87	NR
Tsere DB 2022 (70)	Adult	EPTB	NR	Tanzania	PLWH	Alere LAM	98	MRS (Xpert)	Positivity: MRS+ 95.8, MRS- 16.2%	NR
Acharya S 2022 (71)	Adult	ATB	Out & In	India	PLWH	Alere LAM	128	MRS (Xpert)	Positivity: MRS+ 84.6%, MRS- 43.4%	NR
Huerga H 2023 (72)	Adult	PTB EPTB	Out	Uganda, Kenya Mozambique, South Africa	PLWH	Alere LAM<br>Fuji LAM	1106<br>1261	1. MRS (culture, Xpert)<br>2. CRS	Alere LAM:<br>1. Sen 0.40, Spe 0.86<br>2. Sen 0.38, Spe 0.95<br>Fuji LAM:<br>1. Sen 0.60, Spe 0.87<br>2. Sen 0.48, Spe 0.90	Yes
Bonnet M 2023 (73)	Adult	PTB EPTB	NR	Cote d'Ivoire, Uganda, Cambodia, Vietnam	PLWH	Alere LAM	525	CRS	Positivity: CRS+ 56.2%, CRS- 95.6%	NR
Cummings MJ 2023 (74)	Adult	PTB	In	Uganda	PLWH	Alere LAM	32	MRS (Xpert)	Positivity: MRS+ 63.2%, MRS- 100%	NR
Åhsberg J 2023 (75)	Adult	PTB EPTB	In	Ghana	PLWH	Alere LAM	86	MRS (Xpert)	Positivity: MRS+ 33.3%, MRS- 16.3%	NR
Szekely R 2024 (76)	Adult	ATB	Out & In	Malawi, South Africa, Tanzania, Thailand Uganda, Vietnam, Zambia	PLWH	Fuji LAM	1628	eMRS (culture, Xpert, additional test)	Sen 0.54, Spe 0.85	Yes
Mohapatra A 2024 (77)	Adult	EPTB	NR	India	PLWH Non-HIV	Alere LAM	170	1. MRS (culture, Xpert/TrueNat)<br>2. CRS	1. Sen 0.19, Spe 0.97<br>2. Sen 0.15, Spe 0.98	Yes
Huang Z 2024 (78)	Adult	PTB EPTB	NR	China	Non-HIV	QBs-LAM	169	1. MRS (culture, Xpert)<br>2. CRS	1. Sen 0.50<br>2. Sen 0.52, Spe 0.96	NR
Reeve BWP 2024 (79)	Adult	PTB	Out	South Africa	PLWH	Alere LAM	732	MRS (culture)	Sen 0.14, Spe 0.99	Yes
**Urine LAM immunoassay (e.g., ELISA, CLEIA) (45 publications, 47 tests)**										
Tessema TA 2001 (80)	Adult	PTB	Out	Ethiopia	NR	ELISA	1000	CRS	Sen 0.74, Spe 0.87	NR
Hamasur B 2001 (81)	Adult	PTB EPTB	NR	Ethiopia, Sweden	NR	ELISA	41	MRS (culture, AFB)	Sen 0.93, Spe 0.96	NR
Boehme C 2005 (82)	NR	PTB	NR	Tanzania	PLWH Non-HIV	ELISA	235	MRS (culture)	Sen 0.80, Spe 0.99	NR

Daley P<br>2009 (83)	Adult	PTB<br>EPTB	NR	India	PLWH<br>Non-HIV	ELISA	200	1. MRS (culture)<br>2. CRS	1. Sen 0.18, Spe 0.88<br>2. Sen 0.17, Spe 0.88	NR
Mutetwa R<br>2009 (84)	Adult<br>Adolescent	PTB	Out & In	Zimbabwe	PLWH<br>Non-HIV	ELISA	261	MRS (culture)	Sen 0.44, Spe 0.89	NR
Lawn SD<br>2009 (85)	Adult	PTB	NR	South Africa	PLWH	ELISA	235	MRS (culture)	Unconc: Sen 0.33, Spe 1.0<br>Conc: Sen 0.38, Spe 1.0	NR
Reither K<br>2009 (86)	Adult	PTB	NR	Tanzania	PLWH<br>Non-HIV	ELISA	291	MRS (culture, AFB)	Sen 0.51, Spe 0.88	NR
Shah M<br>2009 (87)	Adult	PTB<br>EPTB	In	South Africa	PLWH<br>Non-HIV	ELISA (Clearview)	155	MRS (culture, AFB)	Sen 0.59, Spe 0.96	NR
Dheda K<br>2010 (88)	Adult	PTB	Out	South Africa	PLWH<br>Non-HIV	ELISA (Clearview)	313	MRS (culture)	Sen 0.13, Spe 0.99	NR
Shah M<br>2010 (89)	Adult	PTB<br>EPTB	In	South Africa	PLWH<br>Non-HIV	ELISA (Clearview)	499	MRS (culture, AFB)	Sen 0.59, Spe 0.96	NR
Gounder CR<br>2011 (90)	Adult	PTB<br>EPTB	Out	South Africa	PLWH	ELISA (Clearview)	414	MRS (culture, AFB)	Sen 0.32, Spe 0.98	NR
Peter JG<br>2012 (1)	Adult	PTB<br>EPTB	In	South Africa	PLWH	ELISA (Clearview)	423	1. MRS (culture)<br>2. CRS	1. Sen 0.59, Spe 0.80<br>2. Sen 0.51, Spe 0.96	Yes
Lawn SD<br>2012 (2)	Adult	PTB	Out	South Africa	PLWH	ELISA (Clearview)	516	MRS (culture)	Sen 0.27, Spe 0.98	Yes
Elsawy A<br>2012 (91)	NR	PTB	NR	Egypt	NR	ELISA (Clearview)	72	MRS (culture)	Sen 0.94, Spe 1.0	NR
Lawn SD<br>2012 (3)	Adult	PTB	Out	South Africa	PLWH	ELISA (Clearview)	84	MRS (culture)	Sen 0.27	NR
Wood R<br>2012 (92)	Adult	PTB<br>EPTB	Out	South Africa	PLWH<br>Non-HIV	ELISA (Clearview)	119	MRS (culture, AFB)	Positivity: MRS+ 24.4%	NR
Talbot E<br>2012 (93)	Adult	PTB	In	Tanzania	PLWH	ELISA (Clearview)	212	MRS (culture)	Sen 0.65, Spe 0.86	Yes
Savolainen L<br>2013 (94)	Adult	PTB<br>EPTB<br>DTB	NR	Finland, Taiwan	Non-HIV	ELISA (Clearview)	129	MRS (culture, AFB)	Unconc: Sen 0.07, Spe 0.97<br>Conc: Sen 0.57, Spe 0.89	NR
Nicol MP<br>2014 (8)	Children	PTB	NR	South Africa	PLWH<br>Non-HIV	ELISA (Clearview)	535	MRS (culture)	Sen 0.02, Spe 0.96	NR
Hamasur B<br>2015 (95)	Adult	PTB<br>EPTB	Out	Sweden	Non-HIV	Uri-TB-direct	39	MRS (culture, PCR)	Sen 0.82, Spe 1.0	NR
Kroidl I<br>2015 (13)	Children	PTB	Out	Tanzania	PLWH<br>Non-HIV	ELISA	53<br>89	1. MRS (culture, Xpert)<br>2. CRS	1. Sen 0.44, Spe 0.97<br>2. Sen 0.26, Spe 0.97	NR
Chan CE<br>2015 (96)	Adult	PTB	NR	Georgia	Non-HIV	ELISA	60	MRS (culture, AFB)	Sen 0.40, Spe 0.92	NR
Hanifa Y<br>2015 (97)	NR	PTB	Prison	South Africa	PLWH<br>Non-HIV	ELISA (Clearview)	844	MRS (culture)	Sen 0.03, Spe 1.0	NR
Cox JA<br>2015 (17)	Adult	UGTB	NA<br>(Autopsy)	Uganda	PLWH	ELISA (Clearview)	36	CRS (AFB,<br>histopathology)	Sen 0.63, Spe 1.0	NR
Iskandar A<br>2017 (98)	Children	PTB<br>EPTB	NR	Indonesia	PLWH<br>Non-HIV	ELISA	61	1. MRS (AFB, culture)<br>2. CRS	1. Sen 0.33, Spe 0.60<br>2. Sen 0.86, Spe 0.83	NR
Amin AG<br>2018 (99)	Adult	PTB	Out	Vietnam, South<br>Africa, Peru	PLWH<br>Non-HIV	ELISA	100	MRS (culture)	Sen 0.98, Spe 0.92	Yes
Dahiya B<br>2019 (100)	Adult	PTB<br>EPTB	Out	India	Non-HIV	ELISA	243	CRS	PTB: Sen 0.50, Spe 0.94<br>EPTB: Sen 0.45, Spe 0.95	NR
Younis H<br>2019 (35)	Adult	PTB	NR	South Africa	PLWH	ELISA (Clearview)	104	MRS (culture, Xpert)	Sen 0.28, Spe 1.0	Yes

Mthiyane T 2019 (101)	NR	PTB	In	South Africa	PLWH	ELISA (Clearview)	156	MRS (culture)	Sen 0.56, Spe 0.78	NR
Wood R 2019 (102)	Adult	PTB	Out & In	Uganda	Non-HIV	ELISA<br>SM FLISA	20	MRS (culture, Xpert)	ELISA: Sen NA<br>SM-FLISA: Sen 0.95, Spe 1.0	NR
Sigal GB 2019 (103)	Adult	PTB	NR	Bangladesh, Peru, South Africa, Vietnam	PLWH<br>Non-HIV	ECL	75	MRS (culture, AFB)	Sen 0.93, Spe 0.97	NR
Broger T 2019 (104)	Adult	PTB	NR	Bangladesh, Peru, South Africa, Vietnam	PLWH<br>Non-HIV	ECL	75	MRS (culture)	Sen 0.93, Spe 0.97	NR
Magni R 2020 (105)	Adult<br>Children	PTB<br>EPTB	NR	Uganda, Peru, Venezuela, Guinea Bissau	PLWH<br>Non-HIV	Immunoassay	430	MRS (culture, Xpert, AFB)	Sen 0.90, Spe 0.74, AUC 0.90	NR
Broger T 2020 (44)	Adult	PTB	Out	South Africa, Peru	Non-HIV	ECL	372	MRS (culture, Xpert)	Sen 0.67, Spe 0.98	Yes
Amin AG 2021 (106)	Adult<br>Children	PTB	NR	Peru, South Africa	Non-HIV	ELISA	160	MRS (culture)	Sen 0.91–0.99, Spe>0.99	NR
Panraksa Y 2021 (107)	Adult	PTB	Out	Vietnam, South Africa, Peru	PLWH<br>Non-HIV	ELISA	25	MRS (culture)	Positivity: MRS+ 100%, Non-TB 0%	NR
Amin AG 2022 (108)	Children	PTB	NR	Peru	NR	ELISA	91	CRS	AUC 0.95	NR
Huang H 2023 (109)	NR	PTB<br>EPTB	NR	China	PLWH<br>Non-HIV	ConA-based ELISA	71	MRS (culture, Xpert, AFB)	TB+/HIV+: Sen 0.44, Spe 1.0<br>TB+/HIV-: Sen 0.38, Spe 1.0	NR
Huang H 2023 (110)	NR	PTB<br>EPTB	NR	China	Non-HIV	CLEIA	235	CRS	Sen 0.51, Spe 0.96	NR
Kamra E 2023 (111)	NR	UGTB	NR	India	Non-HIV	Magneto-ELISA	82	1. MRS (culure, Xpert, AFB)<br>2. CRS	1. Sen 0.71, Spe 0.91<br>2. Sen 0.60, Spe 0.91	NR
Zhang Y 2023 (112)	Adult	PTB	NR	China	Non-HIV	CLEIA	216	CRS	Sen 0.52, Spe 0.98	Yes
Gao M 2024 (113)	Adult	PTB<br>EPTB	In	China	Non-HIV	CLEIA	252	CRS	PTB: Sen 0.55, Spe 1.0<br>EPTB: Sen 0.40, Spe 1.0	NR
Ajantha P 2024 (114)	Adult	PTB	NR	India	Non-HIV	ELISA	80	MRS (culture, Xpert)	Sen 0.89–1.0, Spe 0.92–1.0	NR
Li Y 2024 (115)	NR	ATB	NR	China	Non-HIV	CLEIA	191	CRS	Sen 0.55, Spe 1.0	NR
Junpeng Z 2024 (116)	NR	ATB	In	Malaysia	PLWH<br>Non-HIV	ELISA (completion, sandwich)	306	CRS	Competetion ELISA: Sen 0.19, Spe 0.98<br>Sandwich ELISA: Sen 0.59, Spe 0.83	NR
**Urine LAM: Other assays (2 publications, 2 tests)**										
Paris L 2017 (117)	NR	PTB	In	Peru	Non-HIV	Nanocage technology	101	MRS (culture)	Sen >0.95, Spe >0.8	NR
Dahiya B 2019 (100)	Adult	PTB<br>EPTB	Out	India	Non-HIV	I-PCR	243	CRS	PTB: Sen 0.74, Spe 0.92<br>EPTB: Sen 0.68, Spe 0.93	NR
**Other urine-based, pathogen-derived biomarkers (19 publications, 26 tests, 30 markers)**										
Bentley-Hibbert SI 1999 (118)	NR	PTB<br>EPTB	NR	US	PLWH<br>Non-HIV	Ag85B, Immunodotblot	56	PTB: MRS (culture, AFB, Xpert)<br>EPTB: CRS	No statistical difference in Ag85 means between any group.	NR
Choudhry V 2002 (119)	NR	PTB	NR	India	NR	CFP, ELISA	54	CRS	Positivity：CRS+ 75%	NR

Singh KK 2003 (120)	NR	ATB	NR	India, US, Cameroon	PLWH Non-HIV	CFP; MPT32, ELISA	43	MRS (culture, AFB)	CFP: Sen 0.55, Spe 1.0 MPT32: Sen 0.64, Spe 1.0	NR
Napolitano DR 2008 (121)	Adult	PTB	NR	Brazil, Jamaica	NR	Ornithine carboamyltransferase (Rv1656), ELISA	32	MRS (culture)	Positivity: MRS+ 37.5%, Healthy 0%	NR
Hong SC 2011 (122)	NR	ATB	NR	South Korea	NR	CFP-10, SPR	55	MRS (AFB)	Positivity: MRS+ 100%, Healthy 0%	NR
Tamada Y 2012 (123)	Adult	PTB	NR	Japan	NR	MPB64, Dot-blot assay	48	MRS (culture, AFB)	Sen 0.75, Spe 0.85	NR
Pollock NR 2013 (124)	Adult	PTB	NR	US, Mexico	NR	Rv1681, ELISA	96	MRS (culture)	Positivity: MRS+ 44%, NonTB/Healthy 0–4.8%	NR
Turbawaty DK 2017 (125)	Adult	PTB	Out	Indonesia	PLWH Non-HIV	ESAT-6/CFP-10/MPT64, ICT	141	MRS (culture)	Sen 0.71, Spe 0.35	NR
Russell TM 2017 (126)	NR	ATB	NR	NR	NR	CFP-10, SOMAmer-	55	MRS (culture)	MRS+ vs. MRS- (p< 0.0276)	NR
Phan LMT 2018 (127)	NR	ATB	NR	NR	NR	CFP-10, Dot-plot assay	46	MRS (culture)	ATB vs. LTBI (p<0.01)	NR
Dahiya B 2019 (100)	Adult	PTB EPTB	Out	India	Non-HIV	CFP-10; ELISA, I-PCR	243	CRS	ELISA: PTB Sen 0.32, EPTB Sen 0.28, Spe 0.93–0.96 I-PCR: PTB Sen 0.49, EPTB Sen 0.45, Spe 0.89–0.94	NR
Broger T 2019 (104)	Adult	PTB	NR	Bangladesh, Peru, South Africa, Vietnam	PLWH Non-HIV	ESAT-6, ECL	75	MRS (culture)	Sen 0.65, Spe 0.97	NR
Phan LMT 2020 (128)	NR	PTB EPTB	NR	South Korea	NR	Ag85B; CFP-10, Immunoblotting	52	MRS (culture)	Ag85B: Sen 0.91, Spe 0.87 CFP-10: Sen 0.76, Spe 0.67	NR
Kim J 2021 (129)	NR	PTB EPTB	NR	South Korea	NR	Ag85B; CFP-10, MagPlas ELISA	222 197	PTB: MRS (culture, Xpert) EPTB: CRS	Ag85B: PTB: Sen 0.47, Spe 0.93 EPTB: Sen 0.31, Spe 0.81 CFP-10: PTB: Sen 0.85, Spe 0.97 EPTB: Sen 0.53, Spe 0.97	NR
Iskandar A 2021 (130)	Children	PTB EPTB	NR	Indonesia	NR	CFP-10, ELISA	78	1. MRS (culture, AFB) 2. CRS	1. Sen 0.61, Spe 0.62 2. Sen 0.65, Spe 0.67	NR
Iskandar A 2020 (131)	Children	PTB	NR	Indonesia	NR	ESAT-6, ELISA	61	1. MRS (culture, AFB) 2. CRS	1. Sen 0.65, Spe 0.67 2. Sen 0.72, Spe 0.67	NR
Dass M 2023 (132)	NR	EPTB	In	India	PLWH Non-HIV	MPT51; MPT64, ELISA	137	CRS	MPT51: Definite Sen 0.70, Probable Sen 0.33, Spe 0.86 MPT64: Definite Sen 0.90, Probable Sen 0.31, Spe 0.92	Yes
Kamra E 2023 (111)	NR	UGTB	NR	India	Non-HIV	LAM/MPT64, MB-AuNP-I-PCR; I-PCR; Magneto-ELISA	82	1. MRS (culure, Xpert, AFB) 2. CRS	MB-AuNP-I-PCR: 1. Sen 1.0, Spe 0.97 2. Sen 0.85, Spe 0.97 I-PCR: 1. Sen 0.86, Spe 0.91 2. Sen 0.65, Spe 0.91 Magneto-ELISA: 1. Sen 0.71, Spe 0.91 2. Sen 0.60, Spe 0.91	NR
Turbawaty DK	Adult	PTB EPTB	Out & In	Indonesia	PLWH Non-HIV	ESAT-6/CFP-10/MPT64, ICT	60	PTB: MRS (AFB) EPTB: CRS	Positivity: TB+/HIV+ 93.3%, TB+/HIV- 100%	NR

2023 (133)									TB+/HIV+ (96.69 ng/mL) vs. TB+ /HIV- (137.73 ng/mL), P=0.001	
**Urine Xpert MTB/RIF assays (28 publications, 29 tests)**										
Hillemann D 2011 (134)	NR	EPTB	NR	Germany	NR	Xpert MTB/RIF	91	MRS (urine culture)	Sen 1.0, Spe 0.99	NR
Tortoli E 2012 (135)	Adult Children	EPTB	NR	Italy	NR	Xpert MTB/RIF	130	1. MRS (urine culture)<br>2. CRS	1. Sen 0.85, Spe 0.97<br>2. Sen 0.88, Spe 0.99	NR
Lawn SD 2012 (3)	Adult	PTB	Out	South Africa	PLWH	Xpert MTB/RIF	84	MRS (culture)	Sen 0.19	NR
Shenai S 2013 (136)	Adult	PTB	NR	South Africa, South Korea	Non-HIV	Xpert MTB/RIF	26	MRS (culture)	Sen 0.04	NR
Lawn SD 2013 (4)	Adult	PTB	Out	South Africa	PLWH	Xpert MTB/RIF	81	MRS (culture)	Sen 0.19, Spe 1.0	NR
Theron G 2014 (137)	NR	PTB EPTB	In	South Africa	PLWH Non-HIV	Xpert MTB/RIF	173	MRS (culture)	Sen NA, Spe 0.82	NR
Sharma SK 2014 (138)	Adult	EPTB	Out & In	India	NR	Xpert MTB/RIF	55	1. MRS (culture)<br>2. CRS	1. Sen 0.33, Spe 1.0<br>2. Sen 0.09–0.33, Spe 1.0	NR
Mazzola E 2016 (139)	NR	PTB EPTB	NR	Italy	NR	Xpert MTB/RIF	235	MRS (urine culture)	Sen 0.88, Spe 1.0	NR
Lawn SD 2017 (24)	Adult	PTB EPTB	In	South Africa	PLWH	Xpert MTB/RIF	139	MRS (sputum/blood culture)	Positivity: Unconc 42.4%, Conc 59.0%	Yes
Pang Y 2017 (140)	NR	UGTB	NR	China	NR	Xpert MTB/RIF	163	1. MRS (urine culture)<br>2. CRS	1. Sen 0.95, Spe 0.87<br>2. Sen 0.63, Spe 0.99	NR
Kerkhoff AD 2017 (29)	Adult	DTB	In	South Africa	PLWH	Xpert MTB/RIF	132	MRS (blood culture)	Positivity: Unconc: 53.7%, Conc: 78%	NR
Samuel BP 2018 (141)	Adult	UGTB	NR	India	PLWH Non-HIV	Xpert MTB/RIF	100	1. MRS (culture)<br>2. CRS	1. Sen 0.90, Spe 0.86<br>2. Sen 0.69, Spe 1.0	NR
Habous M 2019 (142)	NR	EPTB	Out & In	UAE	NR	Xpert MTB/RIF	22	MRS (urine culture)	Sen 0.86, Spe 1.0	NR
Chen Y 2019 (143)	NR	UGTB	NR	China	Non-HIV	Xpert MTB/RIF	302	1. MRS (urine culture)<br>2. CRS	1. Sen 0.94, Spe 0.90<br>2. Sen 0.41, Spe 1.0	NR
Lopez A 2019 (144)	Children	PTB	Out	Philippines	NR	Xpert MTB/RIF	182	CRS	Positivity: 0%	Yes
Andama A 2020 (40)	Adult	PTB	Out & In	Uganda	PLWH Non-HIV	Xpert MTB/RIF Ultra	357	MRS (sputum culture, Xpert)	Sen 0.17, Spe 0.98	Yes
Cresswell FV 2020 (49)	Adult	EPTB (TBM)	In	Uganda	PLWH	Xpert MTB/RIF Ultra	56	CRS	Positivity: Definite: 40.5%, Probable: 21.1%	NR
Mokaddas E 2021 (145)	NR	EPTB	NR	Kuwait	NR	Xpert MTB/RIF	302	MRS (urine culture)	Sen 0.89, Spe 1.0	NR
Song R 2021 (146)	Children	PTB EPTB	Out & In	Kenya	PLWH Non-HIV	Xpert MTB/RIF	31	MRS (culture)	Sen 0.13	Yes
Liu P 2021 (147)	Adult	UGTB	In	China	NR	Xpert MTB/RIF	112	CRS	Sen 0.88, Spe 0.97	NR
Minnies S 2021 (148)	Adult	EPTB	Out	South Africa	PLWH Non-HIV	Xpert MTB/RIF Ultra	135	CRS (culture, histopathology)	Sen 0.18, Spe 0.98	Yes
Wang Y 2022 (149)	Adult	UGTB	NR	China	Non-HIV	Xpert MTB/RIF	161	CRS	Sen 0.79, Spe 0.69	NR
Wake RM 2022 (69)	Adult	PTB EPTB	Out & In	South Africa	PLWH	Xpert MTB/RIF Ultra	100	MRS (culture)	Sen 0.71, Spe 0.99	NR
Moreto-Planas L 2022 (150)	Children	PTB EPTB	NR	Guinea-Bissau, South Sudan	PLWH Non-HIV	Xpert MTB/RIF Ultra	524	CRS	Sen 0.14, Spe 0.99	NR

Dutschke A 2022 (151)	Adult	PTB	Out	Guinea-Bissau	PLWH	Xpert MTB/RIF	203	MRS (Xpert)	Positivity: MRS+ 16.1%, MRS- 2.9%	NR
Zahid QU 2024 (152)	Adult Children	EPTB	NR	Pakistan	NR	Xpert MTB/RIF	61	MRS (urine culture)	Sen 1.0, Spe 0.97	NR
Reeve BWP 2024 (79)	Adult	PTB	Out	South Africa	PLWH	Xpert MTB/RIF Ultra	732	MRS (culture)	Sen 0.25, Spe 0.99	Yes
Hueda-Zavaleta, M 2024 (153)	Adult	EPTB	NR	Peru	NR	Xpert MTB/RIF Xpert MTB/RIF Ultra	37	MRS (urine culture)	Sen 1.0, Spe 0.94	Yes
**Other urine-based molecular assays (36 publications, 37 tests)**										
Mitarai S 1995 (154)	NR	PTB	Out & In	Japan	NR	PCR	81	CRS	Positivity: PTB 32.8%, Non-TB 12.5%	NR
Gamboa F 1997 (155)	NR	PTB EPTB	NR	Spain	NR	rRNA detected by AMTDT	51	MRS (culture)	Sen 0.60, Spe 1.00	NR
Gamboa F 1998 (156)	NR	EPTB	NR	Spain	NR	LCx assay	69	CRS	Sen 0.70, Spe 1.00	NR
Aceti A 1999 (157)	Adult	PTB	In	Italy	PLWH	Nested PCR	156	MRS (culture, AFB)	Positivity: MRS+TB 100%, NTM 0%, HIV+/Mycobacteria- 1.8%, Healthy 0%	NR
Hemal AK 2000 (158)	Adult Children	UGTB	Out	India	NR	PCR	42	CRS	Sen 0.84, Spe 0.86	NR
Kafwabulula M 2002 (159)	Adult	PTB	Out	Zambia	PLWH Non-HIV	Nested PCR	126	MRS (culture)	Githui method: Sen 0.56, Spe 0.98 Sechi method: Sen 0.29, Spe 0.98	NR
Marei AM 2003 (160)	NR	PTB	NR	Egypt	NR	PCR	22	MRS (culture)	sen 0.67, spe 1.0	NR
Torrea G 2005 (161)	Adult	PTB EPTB	Out & In	Burkina Faso	PLWH Non-HIV	Nested PCR	386	PTB: MRS (culture), CRS EPTB: CRS	PTB: MRS+ Sen 0.41, CRS+ Sen 0.67, EPTB: Sen 0.57, Spe 0.98	NR
Cannas A 2008 (162)	Adult	PTB	NR	Italy	PLWH Non-HIV	PCR	66	MRS (culture)	Positivity: PTB 79.1%, Pulmonary disease/Healthy 0%	NR
Sener AG 2008 (163)	NR	UGTB	NR	Turkey	NR	MTD	180	MRS (culture)	Positivity: MRS+ 100%, MRS- 0%	NR
Garcia-Elorriaga G 2009 (164)	NR	UGTB	NR	Mexico	NR	PCR	20	CRS	Sen 1.0, Spe 0.82	NR
Gopinath K 2009 (165)	NR	PTB	NR	India	NR	PCR	193	MRS (culture, PCR)	Positivity: MRS+TB 52.2%, MRS-TB 28.6%, Healthy 0%	NR
da Cruz HL 2011 (166)	NR	PTB EPTB	In	Brazil	NR	Nested PCR	156	CRS	Sen 0.40, Spe 0.91	NR
Ghaleb K 2013 (167)	NR	UGTB	Out & In	Egypt	NR	PCR	300	CRS	Sen 1.0, Spe 1.0	NR
Khan FUR 2013 (168)	Adult	UGTB	NR	Pakistan	NR	PCR	50	MRS (culture)	Sen 0.89, Spe 0.97	NR
Kim JK 2013 (169)	Adult	UGTB	Out	South Korea	NR	PCR	57	MRS (AFB, culture)	Sen 0.86, Spe 0.94	NR
Heydari AA 2014 (170)	Adult	PTB	Out & In	Iran	NR	PCR	107	MRS (culture)	Sen 0.56, Spe 0.66	NR
Lima JF 2015 (171)	Adult	PTB EPTB	NR	Brazil	Non- HIV	Nested PCR	55 47	CRS	PTB: Sen 0.35, Spe 1.0 EPTB: Sen 0.41, Spe 1.0	NR

Jamshidi Makiani M 2016 (172)	NR	PTB EPTB	NR	Iran	PLWH Non-HIV	Nested PCR	100	CRS	Positivity: CRS+ 28%	NR
Ayatollahi J 2016 (173)	NR	PTB	Out & In	Iran	NR	Nested PCR	90	MRS (culture, AFB)	Sen 0.30, Spe 1.0	NR
Bordelon H 2017 (174)	NR	PTB	NR	Peru, South Africa	PLWH Non-HIV	PCR	126	MRS (culture)	MRS+ TB vs. non-TB: no significant difference Higher DNA levels in TB+/HIV+ vs. TB-/HIV+ (p=0.037)	NR
Santos FCF 2018 (175)	Adult Children	EPTB	NR	Brazil	NR	qPCR	46	CRS	Sen 0.33, Spe 1.0	NR
Patel K 2018 (176)	Adult	PTB	NR	South Africa	PLWH Non-HIV	PCR	412	MRS (culture)	Sen 0.43, Spe 0.89	NR
Chemeda A 2019 (177)	Adult	PTB	NR	Ethiopia	PLWH	PCR	117	MRS (culture)	Sen 0.73, Spe 0.89	NR
Bisognin F 2020 (178)	NR	PTB EPTB	NR	Italy	NR	PCR	20	MRS (urine culture)	Sen 0.80, Spe 1.0	NR
Costa-Lima JFD 2020 (179)	Children	PTB EPTB	Out & In	Brazil	PLWH Non-HIV	Nested PCR	62 65	CRS	PTB: Sen 0.39, Spe 0.98 EPTB: Sen 0.20, Spe 0.98	NR
Mokaddas E 2021 (145)	NR	EPTB	NR	Kuwait	NR	PTec-ET	302	MRS (urine culture)	Sen 0.78, Spe 1.0	NR
Oreskovic A 2021 (180)	Adult	PTB	NR	South Africa	PLWH Non-HIV	PCR	73	MRS (Xpert)	Sen 0.84, Spe 1.0	NR
Liu P 2021 (147)	Adult	UGTB	In	China	NR	qPCR	112	CRS	Sen 0.84, Spe 0.93	NR
Chang A 2022 (181)	NR	PTB	NR	Philippines	NR	Shotgun metagenomic DNA sequencing	58	MRS (culture)	Sen 0.52, Spe 0.62–0.97 AUC 0.6–0.93	NR
Wang Y 2022 (149)	Adult	UGTB	NR	China	NR	PCR	161	CRS	Sen 0.83, Spe 0.39	NR
Kamra E 2022 (182)	Adult	UGTB	NR	India	Non-HIV	M-nested PCR M-PCR	130	CRS	M-nested PCR: Sen 0.83, Spe 0.97–0.98 M-PCR: Sen 0.67, Spe 0.93	NR
Araujo RM 2023 (183)	Adult	EPTB	NR	Brazil	NR	IS6110-LAMP	48	CRS	Sen 0.79, Spe 0.71	NR
Tschan Y 2024 (184)	Adult	PTB	NR	Tanzania	NR	Sequence-specific Mtb-DNA hybridization assay	88	MRS (Xpert)	Sen 0.72, Spe 0.96	Yes
Salazar MP 2024 (185)	NR	PTB EPTB	NR	Brazil	NR	Nested PCR	95	CRS	Sen 0.61, Spe 0.52, AUC 0.55	NR
Gao M 2024 (113)	NR	PTB EPTB	In	China	Non-HIV	TB-RNA testing (simultaneous amplification and testing method)	252	CRS	PTB: Sen 0.27, Spe1.0 EPTB: Sen 0.10, Spe 1.0	NR
**Urine culture and AFB smear (15 publications, 25 tests)**										
Hemal AK 2000 (158)	Adult Children	UGTB	Out	India	NR	AFB Culture (LJ)	42	CRS	Positivity: AFB 23.8%, Culture 31.0%	NR
Marei AM 2003 (160)	NR	ATB	NR	Egypt	NR	AFB	22	MRS (culture)	Sen 0.67, Spe 0.88	NR
Gopinath K 2009 (165)	NR	PTB	NR	India	NR	AFB Culture (LJ, Bactec)	46	MRS (culture, PCR)	Positivity: AFB 6.5%, LJ 10.9%, Bactec 26.1%	NR

Sun L 2010 (186)	NR	UGTB	In	China	NR	Culture (LJ)	60	Histopathology	Sen 0.23, Spe 1.0	NR
Ghaleb K 2013 (167)	NR	UGTB	Out & In	Egypt	NR	AFB<br>Culture (LJ, Bactec)	300	CRS	AFB: Sen 0.25, Spe 1.0<br>LJ: Sen 0.25, Spe 1.0<br>Bactec: Sen 0.38, Spe 1.0	NR
Khan FUR 2013 (168)	Adult	UGTB	NR	Pakistan	NR	AFB	50	MRS (culture)	Sen 0.52, Spe 0.95	NR
Kim JK 2013 (169)	Adult	UGTB	Out	South Korea	NR	AFB<br>Culture	57	CRS	Positivity: AFB 8.8%, Culture 12.2%	NR
Zhao N 2017 (187)	Adult	UGTB	In	China	NR	Culture	92	Histopathology	Sen 0.33, Spe 1.0	NR
Pang Y 2017 (140)	NR	UGTB	NR	China	NR	AFB<br>Culture (LJ)	44	CRS	AFB: Sen 0.19, Spe 0.99<br>Culture: Sen 0.46, Spe 1.0	NR
Samuel BP 2018 (141)	Adult	UGTB	NR	India	PLWH<br>Non-HIV	AFB<br>Culture (MGIT)	100	CRS	AFB: Sen 0.33, Spe 1.0<br>Culture: Sen 0.56, Spe 1.0	NR
Habous M 2019 (142)	NR	EPTB	Out & In	UAE	NR	AFB	22	MRS (culture)	Sen 0.57, Spe 1.0	NR
Chen Y 2019 (143)	NR	UGTB	NR	China	Non-HIV	AFB<br>Culture (MGIT)	150	CRS	AFB: Sen 0.07, Spe 0.98<br>Culture: Sen 0.24, Spe 1.0	NR
Costa-Lima JFD 2020 (179)	Children	PTB<br>EPTB	Out & In	Brazil	PLHIV<br>Non-HIV	Culture (LJ)	58	CRS	Culture: Sen 0.07	NR
Liu P 2021 (147)	NR	UGTB	In	China	NR	AFB	83	CRS	Positivity: AFB 25%	NR
Wang Y 2022 (149)	Adult	UGTB	NR	China	Non-HIV	AFB<br>Culture (MGIT)	119<br>33	CRS	AFB: Sen 0.78, Spe 0.34<br>Culture: Sen 0.75, Spe 0.28	NR
**Other urine-based, pathogen-derived assays**										
Marei AM 2003 (160)	NR	ATB	NR	Egypt	NR	FASTPlaque TB	22	MRS (culture)	Sen 1.0, Spe 1.0	NR
**Urine-based host-derived biomarker assays (21 studies)**										
Schon T 1999 (188)	Adult	PTB	NR	Ethiopia	PLWH<br>Non-HIV	Nitric oxide metabolites	47	CRS	Higher levels in HIV-/PTB vs. controls (p<0.01)	NR
Yuksekol I 2003 (189)	Adult	PTB	NR	Turkey	NR	Neopterin	95	CRS	Higher levels in PTB vs. controls (p<0.001)	NR
Young BL 2014 (190)	NR	PTB	Out	South Africa	Non-HIV	Proteomics	63	MRS (culture, AFB)	Higher levels in PTB vs. Non-TB disease (p < 0.05)	NR
Petrone L 2015 (191)	Children	PTB<br>EPTB	NR	Uganda	PLWH<br>Non-HIV	IP-10, ELISA	161	CRS	Non-HIV: Sen 0.53, Spe 0.66<br>PLWH: Sen 0.54, Spe 0.61	NR
Lim SH 2016 (192)	Adult	PTB<br>EPTB	In	US	PLWH<br>Non-HIV	Volatile organic compounds	63	MRS (culture)	Sen 0.86, Spe 0.80	NR
Sandlund J 2018 (193)	Adult	PTB	Out	Kenya	PLWH<br>Non-HIV	Volatile organic compounds	237<br>161	MRS (culture, Xpert)	HIV+: Sen 0.48, Spe 0.62<br>HIV-: Sen 0.78, Spe 0.69	NR
Wang J 2018 (194)	Adult	PTB	NR	China	NR	Proteomics	145	MRS (culture)	Sen 0.86, Spe 0.88, AUC 0.926	NR
Isa F 2018 (195)	Adult	PTB	NR	Haiti, Vietnam	PLWH<br>Non-HIV	Metabolomics	100	MRS (culture)	AUCs>0.80	NR
Kim SY 2018 (196)	Adult	PTB	NR	South Korea	NR	IP-10, ELISA	44	CRS	TB vs. Healthy (p=0.1519)	NR
Fitzgerald BL 2019 (197)	Adult	ATB	NR	Uganda, South Africa	NR	Metabolomics (SLC1G)	85	MRS (culture)	Higher levels in TB vs. controls (p<0.0001)	NR
Petrone L 2019 (198)	NR	PTB<br>EPTB	NR	Italy	NR	IP-10, ELISA	58	CRS	Higher levels in TB vs. healthy control (p<0.0001)	Yes

Izquierdo-Garcia JL 2020 (199)	Adult	PTB EPTB	NR	Spain	NR	Metabolomics	229	MRS (culture)	Sen 1.0, Spe 1.0	NR
Eribo OA 2020 (200)	Adult	ATB	Out	South Africa	PLWH Non-HIV	Host biomarkers	151	MRS (culture)	HIV+: sIL6R/MMP-9/IL-2Ra/IFN-γ Sen 0.86, Spe 0.95, AUC 0.96 HIV-: sIL6R/sIL-2Ra Sen 0.54, Spe 0.80, AUC 0.76	NR
Deng J 2021 (201)	Adult	PTB	NR	China	NR	Metabolomics	90	CRS	GSH: AUC 0.76–0.98 Histamine: AUC 0.88–1.0	NR
Liu L 2021 (202)	Adult	PTB	NR	China	NR	Proteomics	156	CRS	Sen 0.83, Spe 0.92	NR
Comella-Del-Barrio P 2021 (203)	Children	ATB	NR	Haiti	NR	Metabolomics	117	CRS	AUC 0.65	NR
Elvira D 2021 (204)	Adult	ATB	NR	Indonesia	PLWH	IP-10, ELISA	30	CRS	TB/HIV (32.9 ± 26.2 pg/mL) vs.Healthy (17 pg/mL).	NR
Kaushik A 2022 (205)	NR	PTB	NR	India	NR	Proteomics	56	MRS (culture)	Higher LRG1, AZGP1 in TB vs, disease/healthy control (p<0.001))	NR
Lyu L 2024 (206)	Adult	PTB	NR	China	Non-HIV	Lipid profile of urinary EVs	41	MRS (culture, AFB)	Sen 0.89, Spe 1.0, AUC 0.82–0.95	NR
Olivier C 2024 (207)	Adult	ATB	NR	South Africa	PLWH Non-HIV	Metabolomics	91	MRS (Xpert)	TB patients showed distinct metabolic disruptions compared to healthy controls.	NR
Isaiah S 2024 (208)	Children	EPTB (TBM)	In	South Africa	Non-HIV	Metabolomics	75	CRS	Metabolomic profiling showed distinct differences between TBM vs. controls (p<0.01)	NR

Abbreviations

In; Inpatient, Out: Outpatient, PTB; Pulmonary tuberculosis, EPTB; Extrapulmonary tuberculosis, ATB; Active tuberculosis, NR; Not reported, ED; Emergency department, ICU; Intensive care unit, UAE; United Arab Emirates, PLWH; People living with HIV, AFB; Acid-fast bacilli, MRS; Microbiological reference standard, CRS; Composite reference standard, Sen; Sensitivity, Spe; Specificity, Alere LAM; Determine™ TB LAM Ag, Fuji LAM; FUJIFILM SILVAMP TB LAM, LFA; Lateral flow assay, QBs; Quantum dot nanobeads, ELISA; Enzyme-linked immunosorbent assay, Clearview; Clearview™ TB ELISA, SM FLISA; Silver-Enhanced Multicolour Fluorescence-Linked Immunosorbent Assay, ECL; Electrochemiluminescence, CLEIA; Chemiluminescent Enzyme Immunoassay, I-PCR; Immuno-polymerase chain reaction, CFP; Culture Filtrate Protein, MPT; Mycobacterium tuberculosis protein antigen, SPR; Surface plasmon resonance, MPB; Mycobacterium bovis BCG Protein, ESAT-6; Early Secreted Antigenic Target of 6 kDa, ICT; Immunochromatographic Test, MB-AuNP-I-PCR; magnetic bead-coupled gold nanoparticle-based I-PCR, PCR; Polymerase chain reaction, AMTDT; Amplified mycobacterium tuberculosis direct test, MTD; Gen-probe amplified Mycobacterium tuberculosis direct test, PTec-ET; ProbeTec ET assay, M-nested PCR; Multiplex nested PCR, M-PCR; Multiplex PCR, LAMP; Loop-Mediated Isothermal Amplification, LJ; Lowenstein–Jensen culture, MGIT; Mycobacterial growth indicator tube, TBM; Tuberculosis meningitis, DTB; Disseminated TB, ITTB: Intrathorathic TB, LNTB: Lymph node tuberculosis

Reference list for supplementary 3

1. Peter JG, Theron G, Zyl-Smit R van, Haripersad A, Mottay L, Kraus S, et al. Diagnostic accuracy of a urine lipoarabinomannan strip-test for TB detection in HIV-infected hospitalised patients. European Respiratory Journal. 2012 Nov 1;40(5):1211–20.

2. Lawn SD, Kerkhoff AD, Vogt M, Wood R. Diagnostic accuracy of a low-cost, urine antigen, point-of-care screening assay for HIV-associated pulmonary tuberculosis before antiretroviral therapy: a descriptive study. Lancet Infect Dis. 2012 Mar;12(3):201–9.

3. Lawn SD, Kerkhoff AD, Vogt M, Wood R. High diagnostic yield of tuberculosis from screening urine samples from HIV-infected patients with advanced immunodeficiency using the Xpert MTB/RIF assay. J Acquir Immune Defic Syndr. 2012 July 1;60(3):289–94.

4. Lawn SD, Kerkhoff AD, Vogt M, Wood R. HIV-associated tuberculosis: relationship between disease severity and the sensitivity of new sputum-based and urine-based diagnostic assays. BMC Med. 2013 Oct 29;11:231.

5. Peter JG, Theron G, Dheda K. Can point-of-care urine LAM strip testing for tuberculosis add value to clinical decision making in hospitalised HIV-infected persons? PLoS One. 2013;8(2):e54875.

6. Nakiyingi L, Moodley VM, Manabe YC, Nicol MP, Holshouser M, Armstrong DT, et al. Diagnostic accuracy of a rapid urine lipoarabinomannan test for tuberculosis in HIV-infected adults. J Acquir Immune Defic Syndr. 2014 July 1;66(3):270–9.

7. Balcha TT, Winqvist N, Sturegård E, Skogmar S, Reepalu A, Jemal ZH, et al. Detection of lipoarabinomannan in urine for identification of active tuberculosis among HIV-positive adults in Ethiopian health centres. Trop Med Int Health. 2014 June;19(6):734–42.

8. Nicol MP, Allen V, Workman L, Isaacs W, Munro J, Pienaar S, et al. Urine lipoarabinomannan testing for diagnosis of pulmonary tuberculosis in children: a prospective study. Lancet Glob Health. 2014 May;2(5):e278-284.

9. Drain PK, Losina E, Coleman SM, Giddy J, Ross D, Katz JN, et al. Diagnostic accuracy of a point-of-care urine test for tuberculosis screening among newly-diagnosed HIV-infected adults: a prospective, clinic-based study. BMC Infect Dis. 2014 Feb 26;14:110.

10. Kerkhoff AD, Wood R, Vogt M, Lawn SD. Predictive value of anemia for tuberculosis in HIV-infected patients in Sub-Saharan Africa: an indication for routine microbiological investigation using new rapid assays. J Acquir Immune Defic Syndr. 2014 May 1;66(1):33–40.

11. Manabe YC, Nonyane BAS, Nakiyingi L, Mbabazi O, Lubega G, Shah M, et al. Point-of-care lateral flow assays for tuberculosis and cryptococcal antigenuria predict death in HIV infected adults in Uganda. PLoS One. 2014;9(7):e101459.

12. Bjerrum S, Kenu E, Lartey M, Newman MJ, Addo KK, Andersen AB, et al. Diagnostic accuracy of the rapid urine lipoarabinomannan test for pulmonary tuberculosis among HIV-infected adults in Ghana-findings from the DETECT HIV-TB study. BMC Infect Dis. 2015 Oct 1;15:407.

13. Kroidl I, Clowes P, Reither K, Mtafya B, Rojas-Ponce G, Ntinginya EN, et al. Performance of urine lipoarabinomannan assays for paediatric tuberculosis in Tanzania. Eur Respir J. 2015 Sept;46(3):761–70.

14. Nakiyingi L, Nonyane BAS, Ssengooba W, Kirenga BJ, Nakanjako D, Lubega G, et al. Predictors for MTB Culture-Positivity among HIV-Infected Smear-Negative Presumptive Tuberculosis Patients in Uganda: Application of New Tuberculosis Diagnostic Technology. PLoS One. 2015;10(7):e0133756.

15. Peter J, Theron G, Chanda D, Clowes P, Rachow A, Lesosky M, et al. Test characteristics and potential impact of the urine LAM lateral flow assay in HIV-infected outpatients under investigation for TB and able to self-expectorate sputum for diagnostic testing. BMC Infect Dis. 2015 July 9;15:262.

16. Drain PK, Losina E, Coleman SM, Giddy J, Ross D, Katz JN, et al. Value of urine lipoarabinomannan grade and second test for optimizing clinic-based screening for HIV-associated pulmonary tuberculosis. J Acquir Immune Defic Syndr. 2015 Mar 1;68(3):274–80.

17. Cox JA, Lukande RL, Kalungi S, Van Marck E, Van de Vijver K, Kambugu A, et al. Is Urinary Lipoarabinomannan the Result of Renal Tuberculosis? Assessment of the Renal Histology in an Autopsy Cohort of Ugandan HIV-Infected Adults. PLoS One. 2015;10(4):e0123323.

18. Hanifa Y, Fielding KL, Chihota VN, Adonis L, Charalambous S, Karstaedt A, et al. Diagnostic Accuracy of Lateral Flow Urine LAM Assay for TB Screening of Adults with Advanced Immunosuppression Attending Routine HIV Care in South Africa. PLoS One. 2016;11(6):e0156866.

19. Drain PK, Losina E, Coleman SM, Giddy J, Ross D, Katz JN, et al. Rapid urine lipoarabinomannan assay as a clinic-based screening test for active tuberculosis at HIV diagnosis. BMC Pulm Med. 2016 Nov 14;16(1):147.

20. Drain PK, Gounder L, Sahid F, Moosa MYS. Rapid Urine LAM Testing Improves Diagnosis of Expectorated Smear-Negative Pulmonary Tuberculosis in an HIV-endemic Region. Sci Rep. 2016 Feb 11;6:19992.

21. Zijenah LS, Kadzirange G, Bandason T, Chipiti MM, Gwambiwa B, Makoga F, et al. Comparative performance characteristics of the urine lipoarabinomannan strip test and sputum smear microscopy in hospitalized HIV-infected patients with suspected tuberculosis in Harare, Zimbabwe. BMC Infect Dis. 2016 Jan 22;16:20.

22. Peter JG, Zijenah LS, Chanda D, Clowes P, Lesosky M, Gina P, et al. Effect on mortality of point-of-care, urine-based lipoarabinomannan testing to guide tuberculosis treatment initiation in HIV-positive hospital inpatients: a pragmatic, parallel-group, multicountry, open-label, randomised controlled trial. Lancet. 2016 Mar 19;387(10024):1187–97.

23. Suwanpimolkul G, Kawkitinarong K, Manosuthi W, Sophonphan J, Gatechompol S, Ohata PJ, et al. Utility of urine lipoarabinomannan (LAM) in diagnosing tuberculosis and predicting mortality with and without HIV: prospective TB cohort from the Thailand Big City TB Research Network. Int J Infect Dis. 2017 June;59:96–102.

24. Lawn SD, Kerkhoff AD, Burton R, Schutz C, Boulle A, Vogt M, et al. Diagnostic accuracy, incremental yield and prognostic value of Determine TB-LAM for routine diagnostic testing for tuberculosis in HIV-infected patients requiring acute hospital admission in South Africa: a prospective cohort. BMC Med. 2017 Mar 21;15(1):67.

25. Gina P, Randall PJ, Muchinga TE, Pooran A, Meldau R, Peter JG, et al. Early morning urine collection to improve urinary lateral flow LAM assay sensitivity in hospitalised patients with HIV-TB co-infection. BMC Infect Dis. 2017 May 12;17(1):339.

26. Sahle SN, Asress DT, Tullu KD, Weldemariam AG, Tola HH, Awas YA, et al. Performance of point-of-care urine test in diagnosing tuberculosis suspects with and without HIV infection in selected peripheral health settings of Addis Ababa, Ethiopia. BMC Res Notes. 2017 Jan 31;10(1):74.

27. Floridia M, Ciccacci F, Andreotti M, Hassane A, Sidumo Z, Magid NA, et al. Tuberculosis Case Finding With Combined Rapid Point-of-Care Assays (Xpert MTB/RIF and Determine TB LAM) in HIV-Positive Individuals Starting Antiretroviral Therapy in Mozambique. Clin Infect Dis. 2017 Nov 13;65(11):1878–83.

28. Huerga H, Ferlazzo G, Bevilacqua P, Kirubi B, Ardizzoni E, Wanjala S, et al. Incremental Yield of Including Determine-TB LAM Assay in Diagnostic Algorithms for Hospitalized and Ambulatory HIV-Positive Patients in Kenya. PLoS One. 2017;12(1):e0170976.

29. Kerkhoff AD, Barr DA, Schutz C, Burton R, Nicol MP, Lawn SD, et al. Disseminated tuberculosis among hospitalised HIV patients in South Africa: a common condition that can be rapidly diagnosed using urine-based assays. Sci Rep. 2017 Sept 7;7(1):10931.

30. Boyles TH, Griesel R, Stewart A, Mendelson M, Maartens G. Incremental yield and cost of urine Determine TB-LAM and sputum induction in seriously ill adults with HIV. Int J Infect Dis. 2018 Oct;75:67–73.

31. LaCourse SM, Pavlinac PB, Cranmer LM, Njuguna IN, Mugo C, Gatimu J, et al. Stool Xpert MTB/RIF and urine lipoarabinomannan for the diagnosis of tuberculosis in hospitalized HIV-infected children. AIDS. 2018 Jan 2;32(1):69–78.

32. Gautam H, Singla M, Jain R, Lodha R, Kabra SK, Singh UB. Point-of-care urine lipoarabinomannan antigen detection for diagnosis of tuberculosis in children. Int J Tuberc Lung Dis. 2019 June 1;23(6):714–9.

33. Broger T, Sossen B, du Toit E, Kerkhoff AD, Schutz C, Ivanova Reipold E, et al. Novel lipoarabinomannan point-of-care tuberculosis test for people with HIV: a diagnostic accuracy study. Lancet Infect Dis. 2019 Aug;19(8):852–61.

34. Van Hoving DJ, Lahri S, Lategan HJ, Nicol MP, Maartens G, Meintjes G. Brief Report: Real-World Performance and Interobserver Agreement of Urine Lipoarabinomannan in Diagnosing HIV-Associated Tuberculosis in an Emergency Center. J Acquir Immune Defic Syndr. 2019 May 1;81(1):e10–4.

35. Younis H, Kerschbaumer I, Moon JY, Kim RS, Blanc CJ, Chen T, et al. Combining urine lipoarabinomannan with antibody detection as a simple non-sputum-based screening method for HIV-associated tuberculosis. PLoS One. 2019;14(6):e0218606.

36. Songkhla MN, Tantipong H, Tongsai S, Angkasekwinai N. Lateral Flow Urine Lipoarabinomannan Assay for Diagnosis of Active Tuberculosis in Adults With Human Immunodeficiency Virus Infection: A Prospective Cohort Study. Open Forum Infect Dis. 2019 Apr;6(4):ofz132.

37. Yoon C, Semitala FC, Asege L, Katende J, Mwebe S, Andama AO, et al. Yield and Efficiency of Novel Intensified Tuberculosis Case-Finding Algorithms for People Living with HIV. Am J Respir Crit Care Med. 2019 Mar 1;199(5):643–50.

38. Byashalira K, Mbelele P, Semvua H, Chilongola J, Semvua S, Liyoyo A, et al. Clinical outcomes of new algorithm for diagnosis and treatment of Tuberculosis sepsis in HIV patients. Int J Mycobacteriol. 2019;8(4):313–9.

39. Esmail A, Pooran A, Sabur NF, Fadul M, Brar MS, Oelofse S, et al. An Optimal Diagnostic Strategy for Tuberculosis in Hospitalized HIV-Infected Patients Using GeneXpert MTB/RIF and Alere Determine TB LAM Ag. J Clin Microbiol. 2020 Sept 22;58(10):e01032-20.

40. Andama A, Jaganath D, Crowder R, Asege L, Nakaye M, Katumba D, et al. Accuracy and incremental yield of urine Xpert MTB/RIF Ultra versus Determine TB-LAM for diagnosis of pulmonary tuberculosis. Diagn Microbiol Infect Dis. 2020 Jan;96(1):114892.

41. Kerkhoff AD, Sossen B, Schutz C, Reipold EI, Trollip A, Moreau E, et al. Diagnostic sensitivity of SILVAMP TB-LAM (FujiLAM) point-of-care urine assay for extra-pulmonary tuberculosis in people living with HIV. Eur Respir J. 2020 Feb;55(2):1901259.

42. Pasipamire M, Broughton E, Mkhontfo M, Maphalala G, Simelane-Vilane B, Haumba S. Detecting tuberculosis in pregnant and postpartum women in Eswatini. Afr J Lab Med. 2020;9(1):837.

43. Tlali M, Fielding KL, Karat AS, Hoffmann CJ, Muravha T, Grant AD, et al. Sensitivity of the lateral flow urine lipoarabinomannan assay in ambulant adults with advanced HIV disease: data from the TB Fast Track study. Trans R Soc Trop Med Hyg. 2020 Aug 1;114(8):556–60.

44. Broger T, Nicol MP, Sigal GB, Gotuzzo E, Zimmer AJ, Surtie S, et al. Diagnostic accuracy of 3 urine lipoarabinomannan tuberculosis assays in HIV-negative outpatients. J Clin Invest. 2020 Nov 2;130(11):5756–64.

45. Bjerrum S, Broger T, Székely R, Mitarai S, Opintan JA, Kenu E, et al. Diagnostic Accuracy of a Novel and Rapid Lipoarabinomannan Test for Diagnosing Tuberculosis Among People With Human Immunodeficiency Virus. Open Forum Infect Dis. 2020 Jan;7(1):ofz530.

46. García JI, Meléndez J, Álvarez R, Mejía-Chew C, Kelley HV, Sidiki S, et al. Accuracy of the tuberculosis point-of-care Alere determine lipoarabinomannan antigen diagnostic test using α-mannosidase treated and untreated urine in a cohort of people living with HIV in Guatemala. AIDS Res Ther. 2020 Oct 19;17(1):62.

47. Gupta A, Kumar A, Ashraf AA, Madigubba H, Chawla K. A pilot study to evaluate urine LAM assay for diagnosis of pulmonary tuberculosis among non-HIV patients. Trop Doct. 2020 Oct;50(4):343–6.

48. Grant AD, Charalambous S, Tlali M, Karat AS, Dorman SE, Hoffmann CJ, et al. Algorithm-guided empirical tuberculosis treatment for people with advanced HIV (TB Fast Track): an open-label, cluster-randomised trial. Lancet HIV. 2020 Jan;7(1):e27–37.

49. Cresswell FV, Ellis J, Kagimu E, Bangdiwala AS, Okirwoth M, Mugumya G, et al. Standardized Urine-Based Tuberculosis (TB) Screening With TB-Lipoarabinomannan and Xpert MTB/RIF Ultra in Ugandan Adults With Advanced Human Immunodeficiency Virus Disease and Suspected Meningitis. Open Forum Infect Dis. 2020 Apr;7(4):ofaa100.

50. Huerga H, Rucker SCM, Bastard M, Dimba A, Kamba C, Amoros I, et al. Should Urine-LAM Tests Be Used in TB Symptomatic HIV-Positive Patients When No CD4 Count Is Available? A Prospective Observational Cohort Study From Malawi. J Acquir Immune Defic Syndr. 2020 Jan 1;83(1):24–30.

51. van Hoving DJ, Meintjes G, Maartens G, Kengne AP. A multi-parameter diagnostic clinical decision tree for the rapid diagnosis of tuberculosis in HIV-positive patients presenting to an emergency centre. Wellcome Open Res. 2020;5:72.

52. Comella-Del-Barrio P, Bimba JS, Adelakun R, Kontogianni K, Molina-Moya B, Osazuwa O, et al. Fujifilm SILVAMP TB-LAM for the Diagnosis of Tuberculosis in Nigerian Adults. J Clin Med. 2021 June 6;10(11):2514.

53. Comella-Del-Barrio P, Molina-Moya B, Gautier J, Villar-Hernández R, Doresca MJC, Sallés-Mingels B, et al. Diagnostic Performance of the Fujifilm SILVAMP TB-LAM in Children with Presumptive Tuberculosis. J Clin Med. 2021 Apr 28;10(9):1914.

54. Nicol MP, Schumacher SG, Workman L, Broger T, Baard C, Prins M, et al. Accuracy of a Novel Urine Test, Fujifilm SILVAMP Tuberculosis Lipoarabinomannan, for the Diagnosis of Pulmonary Tuberculosis in Children. Clin Infect Dis. 2021 May 4;72(9):e280–8.

55. Connelly JT, Andama A, Grant BD, Ball A, Mwebe S, Asege L, et al. Field evaluation of a prototype tuberculosis lipoarabinomannan lateral flow assay on HIV-positive and HIV-negative patients. PLoS One. 2021;16(7):e0254156.

56. de Vasconcellos K, Ramjathan P, Singh D. The utility of point-of-care urinary lipoarabinomannan testing for the diagnosis of tuberculosis in critically ill patients: a prospective observational study. BMC Infect Dis. 2021 Mar 19;21(1):281.

57. Nkereuwem E, Togun T, Gomez MP, Székely R, Macé A, Jobe D, et al. Comparing accuracy of lipoarabinomannan urine tests for diagnosis of pulmonary tuberculosis in children from four African countries: a cross-sectional study. Lancet Infect Dis. 2021 Mar;21(3):376–84.

58. Muyoyeta M, Kerkhoff AD, Chilukutu L, Moreau E, Schumacher SG, Ruhwald M. Diagnostic accuracy of a novel point-of-care urine lipoarabinomannan assay for the detection of tuberculosis among adult outpatients in Zambia: a prospective cross-sectional study. Eur Respir J. 2021 Nov;58(5):2003999.

59. Kebede W, Abebe G, Gudina EK, Van Rie A. The value of lateral flow urine lipoarabinomannan assay and empirical treatment in Xpert MTB/RIF ultra negative patients with presumptive TB: a prospective cohort study. Sci Rep. 2021 Dec 24;11(1):24428.

60. Schramm B, Nganaboy RC, Uwiragiye P, Mukeba D, Abdoubara A, Abdou I, et al. Potential value of urine lateral-flow lipoarabinomannan (LAM) test for diagnosing tuberculosis among severely acute malnourished children. PLoS One. 2021;16(5):e0250933.

61. Chernick L, Kalla IS, Venter M. Clinical, radiological, and laboratory predictors of a positive urine lipoarabinomannan test in sputum-scarce and sputum-negative patients with HIV-associated tuberculosis in two Johannesburg hospitals. South Afr J HIV Med. 2021;22(1):1234.

62. Simieneh A, Tadesse M, Kebede W, Gashaw M, Abebe G. Combination of Xpert® MTB/RIF and DetermineTM TB-LAM Ag improves the diagnosis of extrapulmonary tuberculosis at Jimma University Medical Center, Oromia, Ethiopia. PLoS One. 2022;17(2):e0263172.

63. Boloko L, Schutz C, Sibiya N, Balfour A, Ward A, Shey M, et al. Xpert Ultra testing of blood in severe HIV-associated tuberculosis to detect and measure Mycobacterium tuberculosis blood stream infection: a diagnostic and disease biomarker cohort study. Lancet Microbe. 2022 July;3(7):e521–32.

64. Orikiriza P, Smith J, Ssekyanzi B, Nyehangane D, Taremwa IM, Turyashemererwa E, et al. Tuberculosis diagnostic accuracy of stool Xpert MTB/RIF and urine AlereLAM in vulnerable children. European Respiratory Journal [Internet]. 2021 Dec 31 [cited 2024 Dec 13];59(1). Available from: https://publications.ersnet.org/content/erj/59/1/2101116

65. Shapiro AE, Olson AM, Kidoguchi L, Niu X, Ngcobo Z, Magcaba ZP, et al. Complementary Nonsputum Diagnostic Testing for Tuberculosis in People with HIV Using Oral Swab PCR and Urine Lipoarabinomannan Detection. J Clin Microbiol. 2022 Aug 17;60(8):e0043122.

66. Kanyama C, Chagomerana MB, Chawinga C, Ngoma J, Shumba I, Kumwenda W, et al. Implementation of tuberculosis and cryptococcal meningitis rapid diagnostic tests amongst patients with advanced HIV at Kamuzu Central Hospital, Malawi, 2016-2017. BMC Infect Dis. 2022 Mar 5;22(1):224.

67. Indirawati NN, Yunihastuti E, Yulianti M, Nasir UZ, Wulandari D, Rinaldi I. Lateral flow urine lipoarabinomannan assay for extrapulmonary tuberculosis diagnosis in adults who are HIV-positive. Int J Infect Dis. 2022 Sept;122:415–9.

68. Bjerrum S, Åhsberg J, Szekely R, Opintan J, Lartey M, Shah M, et al. Diagnostic Accuracy of Urine Lipoarabinomannan Testing in Early Morning Urine versus Spot Urine for Diagnosis of Tuberculosis among People with HIV. Microbiol Spectr. 2022 Apr 27;10(2):e0020822.

69. Wake RM, Govender NP, Omar SV, Ismail F, Tiemessen CT, Harrison TS, et al. Rapid urine-based screening tests increase the yield of same-day tuberculosis diagnoses among patients living with advanced HIV disease. AIDS. 2022 May 1;36(6):839–44.

70. Tsere DB, Shirima GM, Grundy BS, Heysell SK, Mpagama SG, Mziray SR, et al. Multiple pathogens contribute to human immunodeficiency virus-related sepsis in addition to Mycobacterium tuberculosis: A prospective cohort in Tanzania. Int J Mycobacteriol. 2022;11(3):241–8.

71. Acharya S, Deshpande P, Asirvatham ES, Palkar A, Sarman CJ, Laxmeshwar C, et al. Utility of the lateral flow urine lipoarabinomannan tuberculosis assay in patients with advanced HIV disease at antiretroviral therapy centres in Mumbai, India. PLoS One. 2022;17(9):e0273970.

72. Huerga H, Bastard M, Lubega AV, Akinyi M, Antabak NT, Ohler L, et al. Novel FujiLAM assay to detect tuberculosis in HIV-positive ambulatory patients in four African countries: a diagnostic accuracy study. Lancet Glob Health. 2023 Jan;11(1):e126–35.

73. Bonnet M, Gabillard D, Domoua S, Muzoora C, Messou E, Sovannarith S, et al. High Performance of Systematic Combined Urine Liboarabinomannan Test and Sputum Xpert MTB/RIF for Tuberculosis Screening in Severely Immunosuppressed Ambulatory Adults With. Clin Infect Dis. 2023 July 5;77(1):112–9.

74. Cummings MJ, Bakamutumaho B, Jain K, Price A, Owor N, Kayiwa J, et al. Brief Report: Detection of Urine Lipoarabinomannan Is Associated With Proinflammatory Innate Immune Activation, Impaired Host Defense, and Organ Dysfunction in Adults With Severe HIV-Associated Tuberculosis in Uganda. J Acquir Immune Defic Syndr. 2023 May 1;93(1):79–85.

75. Åhsberg J, Puplampu P, Kwashie A, Commey JO, Ganu VJ, Omari MA, et al. Point-of-Care Urine Lipoarabinomannan Testing to Guide Tuberculosis Treatment Among Severely Ill Inpatients With Human Immunodeficiency Virus in Real-World Practice: A Multicenter Stepped Wedge Cluster-Randomized Trial From Ghana. Clin Infect Dis. 2023 Oct 13;77(8):1185–93.

76. Székely R, Sossen B, Mukoka M, Muyoyeta M, Nakabugo E, Hella J, et al. Prospective multicentre accuracy evaluation of the FUJIFILM SILVAMP TB LAM test for the diagnosis of tuberculosis in people living with HIV demonstrates lot-to-lot variability. PLoS One. 2024;19(5):e0303846.

77. Mohapatra A, Gaikwad U, Ganga RT, Sharma P. Diagnostic accuracy of Lipoarabinomannan detection by lateral flow assay in pleural tuberculosis. BMC Infect Dis. 2024 Feb 9;24(1):178.

78. Huang Z, Huang H, Hu J, Xia L, Liu X, Qu R, et al. A novel quantitative urine LAM antigen strip for point-of-care tuberculosis diagnosis in non-HIV adults. J Infect. 2024 Feb;88(2):194–8.

79. Reeve BWP, Ndlangalavu G, Mishra H, Palmer Z, Tshivhula H, Rockman L, et al. Point-of-care C-reactive protein and Xpert MTB/RIF Ultra for tuberculosis screening and diagnosis in unselected antiretroviral therapy initiators: a prospective, cross-sectional, diagnostic accuracy study. Lancet Glob Health. 2024 May;12(5):e793–803.

80. Tessema TA, Hamasur B, Bjun G, Svenson S, Bjorvatn B. Diagnostic evaluation of urinary lipoarabinomannan at an Ethiopian tuberculosis centre. Scand J Infect Dis. 2001;33(4):279–84.

81. Hamasur B, Bruchfeld J, Haile M, Pawlowski A, Bjorvatn B, Källenius G, et al. Rapid diagnosis of tuberculosis by detection of mycobacterial lipoarabinomannan in urine. J Microbiol Methods. 2001 May;45(1):41–52.

82. Boehme C, Molokova E, Minja F, Geis S, Loscher T, Maboko L, et al. Detection of mycobacterial lipoarabinomannan with an antigen-capture ELISA in unprocessed urine of Tanzanian patients with suspected tuberculosis. Trans R Soc Trop Med Hyg. 2005 Dec;99(12):893–900.

83. Daley P, Michael JS, Hmar P, Latha A, Chordia P, Mathai D, et al. Blinded evaluation of commercial urinary lipoarabinomannan for active tuberculosis: a pilot study. Int J Tuberc Lung Dis. 2009 Aug;13(8):989–95.

84. Mutetwa R, Boehme C, Dimairo M, Bandason T, Munyati SS, Mangwanya D, et al. Diagnostic accuracy of commercial urinary lipoarabinomannan detection in African tuberculosis suspects and patients. Int J Tuberc Lung Dis. 2009 Oct;13(10):1253–9.

85. Lawn SD, Edwards DJ, Kranzer K, Vogt M, Bekker LG, Wood R. Urine lipoarabinomannan assay for tuberculosis screening before antiretroviral therapy diagnostic yield and association with immune reconstitution disease. AIDS. 2009 Sept 10;23(14):1875–80.

86. Reither K, Saathoff E, Jung J, Minja LT, Kroidl I, Saad E, et al. Low sensitivity of a urine LAM-ELISA in the diagnosis of pulmonary tuberculosis. BMC Infect Dis. 2009 Aug 28;9:141.

87. Shah M, Variava E, Holmes CB, Coppin A, Golub JE, McCallum J, et al. Diagnostic accuracy of a urine lipoarabinomannan test for tuberculosis in hospitalized patients in a High HIV prevalence setting. J Acquir Immune Defic Syndr. 2009 Oct 1;52(2):145–51.

88. Dheda K, Davids V, Lenders L, Roberts T, Meldau R, Ling D, et al. Clinical utility of a commercial LAM-ELISA assay for TB diagnosis in HIV-infected patients using urine and sputum samples. PLoS One. 2010 Mar 24;5(3):e9848.

89. Shah M, Martinson NA, Chaisson RE, Martin DJ, Variava E, Dorman SE. Quantitative analysis of a urine-based assay for detection of lipoarabinomannan in patients with tuberculosis. J Clin Microbiol. 2010 Aug;48(8):2972–4.

90. Gounder CR, Kufa T, Wada NI, Mngomezulu V, Charalambous S, Hanifa Y, et al. Diagnostic accuracy of a urine lipoarabinomannan enzyme-linked immunosorbent assay for screening ambulatory HIV-infected persons for tuberculosis. J Acquir Immune Defic Syndr. 2011 Oct 1;58(2):219–23.

91. Elsawy A, Redwan EM. Urine Lipoarabinomannan as Initial Markers for Active Pulmonary Tuberculosis. Aust J Basic & Appl Sci. 2012;6(3):751–5.

92. Wood R, Racow K, Bekker LG, Middelkoop K, Vogt M, Kreiswirth BN, et al. Lipoarabinomannan in urine during tuberculosis treatment: association with host and pathogen factors and mycobacteriuria. BMC Infect Dis. 2012 Feb 27;12:47.

93. Talbot E, Munseri P, Teixeira P, Matee M, Bakari M, Lahey T, et al. Test characteristics of urinary lipoarabinomannan and predictors of mortality among hospitalized HIV-infected tuberculosis suspects in Tanzania. PLoS One. 2012;7(3):e32876.

94. Savolainen L, Kantele A, Sandboge B, Sirén M, Valleala H, Tuompo R, et al. Modification of clearview tuberculosis (TB) enzyme-linked immunosorbent assay for TB patients not infected with HIV. Clin Vaccine Immunol. 2013 Sept;20(9):1479–82.

95. Hamasur B, Bruchfeld J, van Helden P, Källenius G, Svenson S. A sensitive urinary lipoarabinomannan test for tuberculosis. PLoS One. 2015;10(4):e0123457.

96. Chan CE, Götze S, Seah GT, Seeberger PH, Tukvadze N, Wenk MR, et al. The diagnostic targeting of a carbohydrate virulence factor from M.Tuberculosis. Sci Rep. 2015 May 15;5:10281.

97. Hanifa Y, Telisinghe L, Fielding KL, Malden JL, Churchyard GJ, Grant AD, et al. The diagnostic accuracy of urine lipoarabinomannan test for tuberculosis screening in a South African correctional facility. PLoS One. 2015;10(5):e0127956.

98. Iskandar A, Nursiloningrum E, Arthamin MZ, Olivianto E, Chandrakusuma MS. The Diagnostic Value of Urine Lipoarabinomannan (LAM) Antigen in Childhood Tuberculosis. J Clin Diagn Res. 2017 Mar;11(3):EC32–5.

99. Amin AG, De P, Spencer JS, Brennan PJ, Daum J, Andre BG, et al. Detection of lipoarabinomannan in urine and serum of HIV-positive and HIV-negative TB suspects using an improved capture-enzyme linked immuno absorbent assay and gas chromatography/mass spectrometry. Tuberculosis (Edinb). 2018 July;111:178–87.

100. Dahiya B, Khan A, Mor P, Kamra E, Singh N, Gupta KB, et al. Detection of Mycobacterium tuberculosis lipoarabinomannan and CFP-10 (Rv3874) from urinary extracellular vesicles of tuberculosis patients by immuno-PCR. Pathog Dis. 2019 July 1;77(5):ftz049.

101. Mthiyane T, Peter J, Allen J, Connolly C, Davids M, Rustomjee R, et al. Urine lipoarabinomannan (LAM) and antimicrobial usage in seriously-ill HIV-infected patients with sputum smear-negative pulmonary tuberculosis. J Thorac Dis. 2019 Aug;11(8):3505–14.

102. Wood A, Barizuddin S, Darr CM, Mathai CJ, Ball A, Minch K, et al. Ultrasensitive detection of lipoarabinomannan with plasmonic grating biosensors in clinical samples of HIV negative patients with tuberculosis. PLoS One. 2019;14(3):e0214161.

103. Sigal GB, Pinter A, Lowary TL, Kawasaki M, Li A, Mathew A, et al. A Novel Sensitive Immunoassay Targeting the 5-Methylthio-d-Xylofuranose–Lipoarabinomannan Epitope Meets the WHO's Performance Target for Tuberculosis Diagnosis. J Clin Microbiol. 2018 Nov 27;56(12):e01338-18.

104. Broger T, Tsionksy M, Mathew A, Lowary TL, Pinter A, Plisova T, et al. Sensitive electrochemiluminescence (ECL) immunoassays for detecting lipoarabinomannan (LAM) and ESAT-6 in urine and serum from tuberculosis patients. PLoS One. 2019;14(4):e0215443.

105. Magni R, Rruga F, Alsaab FM, Sharif S, Howard M, Espina V, et al. Lipoarabinomannan antigenic epitope differences in tuberculosis disease subtypes. Sci Rep. 2020 Aug 18;10(1):13944.

106. Amin AG, De P, Graham B, Calderon RI, Franke MF, Chatterjee D. Urine lipoarabinomannan in HIV uninfected, smear negative, symptomatic TB patients: effective sample pretreatment for a sensitive immunoassay and mass spectrometry. Sci Rep. 2021 Feb 3;11(1):2922.

107. Panraksa Y, Amin AG, Graham B, Henry CS, Chatterjee D. Immobilization of Proteinase K for urine pretreatment to improve diagnostic accuracy of active tuberculosis. PLoS One. 2021;16(9):e0257615.

108. Amin AG, De P, Graham B, Jensen BL, Moreau E, Chatterjee D. Overcome low levels of detection limit and choice of antibody affects detection of lipoarabinomannan in pediatric tuberculosis. PLoS One. 2022;17(10):e0275838.

109. Huang H, Qu R, Wu K, Xu J, Li J, Lu S, et al. Proteinase K-pretreated ConA-based ELISA assay: a novel urine LAM detection strategy for TB diagnosis. Front Microbiol. 2023;14:1236599.

110. Huang L, Niu Y, Zhang L, Yang R, Wu M. Diagnostic value of chemiluminescence for urinary lipoarabinomannan antigen assay in active tuberculosis: insights from a retrospective study. Front Cell Infect Microbiol. 2023;13:1291974.

111. Kamra E, Prasad T, Rais A, Dahiya B, Sheoran A, Soni A, et al. Diagnosis of genitourinary tuberculosis: detection of mycobacterial lipoarabinomannan and MPT-64 biomarkers within urine extracellular vesicles by nano-based immuno-PCR assay. Sci Rep. 2023 July 18;13(1):11560.

112. Zhang Y, Chen S, Wei H, Zhong Q, Yuan Y, Wang Y, et al. Breakthrough of chemiluminescence-based LAM urine test beyond HIV-positive individuals: Clinical diagnostic value of pulmonary tuberculosis in the general population. Medicine (Baltimore). 2023 Dec 1;102(48):e36371.

113. Gao M, Wu Q, Wang X, Sun X, Li M, Bai G. Advancements in LAM-based diagnostic kit for tuberculosis detection: enhancing TB diagnosis in HIV-negative individuals. Front Microbiol. 2024;15:1367092.

114. Ajantha P, Puri MM, Tayal D, Khalid U. Urinary lipoarabinomannan in individuals with sputum-negative pulmonary tuberculosis. Indian J Med Res. 2024 Feb 1;159(2):206–12.

115. Li Y, Ru Z, Wei H, Wu M, Xie G, Lou J, et al. Improving the diagnosis of active tuberculosis: a novel approach using magnetic particle-based chemiluminescence LAM assay. BMC Pulm Med. 2024 Feb 27;24(1):100.

116. Junpeng Z, Avoi R, Atil A. Analysis of the Validity of Urine LAM ELISA for Tuberculosis Infection. IJPQA. 2024 Mar 25;15(01):258–64.

117. Paris L, Magni R, Zaidi F, Araujo R, Saini N, Harpole M, et al. Urine lipoarabinomannan glycan in HIV-negative patients with pulmonary tuberculosis correlates with disease severity. Sci Transl Med. 2017 Dec 13;9(420):eaal2807.

118. Bentley-Hibbert SI, Quan X, Newman T, Huygen K, Godfrey HP. Pathophysiology of antigen 85 in patients with active tuberculosis: antigen 85 circulates as complexes with fibronectin and immunoglobulin G. Infect Immun. 1999 Feb;67(2):581–8.

119. Choudhry V, Saxena RK. Detection of Mycobacterium tuberculosis antigens in urinary proteins of tuberculosis patients. Eur J Clin Microbiol Infect Dis. 2002 Jan;21(1):1–5.

120. Singh KK, Dong Y, Hinds L, Keen MA, Belisle JT, Zolla-Pazner S, et al. Combined Use of Serum and Urinary Antibody for Diagnosis of Tuberculosis. The Journal of Infectious Diseases. 2003;188(3):371–7.

121. Napolitano DR, Pollock N, Kashino SS, Rodrigues V, Campos-Neto A. Identification of Mycobacterium tuberculosis ornithine carboamyltransferase in urine as a possible molecular marker of active pulmonary tuberculosis. Clin Vaccine Immunol. 2008 Apr;15(4):638–43.

122. Hong SC, Lee J, Shin HC, Kim CM, Park JY, Koh K, et al. Clinical immunosensing of tuberculosis CFP-10 in patient urine by surface plasmon resonance spectroscopy. Sensors and Actuators B: Chemical. 2011 Dec;160(1):1434–8.

123. Tamada Y, Kanda S, Yoshidome A, Hayashi I, Miyake M, Nishiyama T. Diagnosis of active tuberculosis using MPB64, a specific antigen of Mycobacterium bovis. Microbiol Immunol. 2012 Nov;56(11):740–7.

124. Pollock NR, Macovei L, Kanunfre K, Dhiman R, Restrepo BI, Zarate I, et al. Validation of Mycobacterium tuberculosis Rv1681 protein as a diagnostic marker of active pulmonary tuberculosis. J Clin Microbiol. 2013 May;51(5):1367–73.

125. Turbawaty DK, Sugianli AK, Soeroto AY, Setiabudiawan B, Parwati I. Comparison of the Performance of Urinary Mycobacterium tuberculosis Antigens Cocktail (ESAT6, CFP10, and MPT64) with Culture and Microscopy in Pulmonary Tuberculosis Patients. Int J Microbiol. 2017;2017:3259329.

126. Russell TM, Green LS, Rice T, Kruh-Garcia NA, Dobos K, De Groote MA, et al. Potential of High-Affinity, Slow Off-Rate Modified Aptamer Reagents for Mycobacterium tuberculosis Proteins as Tools for Infection Models and Diagnostic Applications. J Clin Microbiol. 2017 Oct;55(10):3072–88.

127. Phan LMT, Rafique R, Baek SH, Nguyen TP, Park KY, Kim EB, et al. Gold-copper nanoshell dot-blot immunoassay for naked-eye sensitive detection of tuberculosis specific CFP-10 antigen. Biosens Bioelectron. 2018 Dec 15;121:111–7.

128. Phan LMT, Kim EB, Cheon SA, Shim TS, Kim HJ, Park TJ. Reliable naked-eye detection of *Mycobacterium tuberculosis* antigen 85B using gold and copper nanoshell-enhanced immunoblotting techniques. Sensors and Actuators B: Chemical. 2020 Aug 15;317:128220.

129. Kim J, Tran VT, Oh S, Jang M, Lee DK, Hong JC, et al. Clinical Trial: Magnetoplasmonic ELISA for Urine-based Active Tuberculosis Detection and Anti-Tuberculosis Therapy Monitoring. ACS Cent Sci. 2021 Nov 24;7(11):1898–907.

130. Iskandar A, Arthamin MZ, Kusdinar G, Handono K, Olivianto E, Aryati A, et al. The diagnostic value of urinary culture filtrate protein-10antigen in childhood tuberculosis. Biomedicine. 2021 Sept 7;41(2):477–81.

131. Iskandar A, Lawanto EM, Zulhaidah M, Olivianto E, Handono K, Anshory M, et al. The Diagnostic Value of Urinary Secretory Antigen Target of 6 kDa in Childhood Pulmonary Tuberculosis. Open Access Macedonian Journal of Medical Sciences. 2020 Dec 26;9(B):841–6.

132. Dass M, Kaur M, Aittan S, Sharma P, Punia S, Muthumohan R, et al. MPT51 and MPT64-based antigen detection assay for the diagnosis of extrapulmonary tuberculosis from urine samples. Diagn Microbiol Infect Dis. 2023 Sept;107(1):115973.

133. Turbawaty DK, Surdjaja NR, Indrati AR, Lismayanti L, Logito V. High Positivity Rate of Urinary Mycobacterium tuberculosis Antigens Cocktail (ESAT-6, CFP-10, and MPT-64) in Active Tuberculosis Patients With and Without Human Immunodeficiency Virus Infection: A Cross-Sectional Study. Clin Pathol. 2023;16:2632010X231198831.

134. Hillemann D, Rüsch-Gerdes S, Boehme C, Richter E. Rapid molecular detection of extrapulmonary tuberculosis by the automated GeneXpert MTB/RIF system. J Clin Microbiol. 2011 Apr;49(4):1202–5.

135. Tortoli E, Russo C, Piersimoni C, Mazzola E, Dal MP, Pascarella M, et al. Clinical validation of Xpert MTB/RIF for the diagnosis of extrapulmonary tuberculosis. European Respiratory Journal [Internet]. 2012 Jan 12 [cited 2024 Dec 29]; Available from: https://publications.ersnet.org/content/erj/early/2012/01/12/09031936.00176311

136. Shenai S, Amisano D, Ronacher K, Kriel M, Banada PP, Song T, et al. Exploring alternative biomaterials for diagnosis of pulmonary tuberculosis in HIV-negative patients by use of the GeneXpert MTB/RIF assay. J Clin Microbiol. 2013 Dec;51(12):4161–6.

137. Theron G, Peter J, Calligaro G, Meldau R, Hanrahan C, Khalfey H, et al. Determinants of PCR performance (Xpert MTB/RIF), including bacterial load and inhibition, for TB diagnosis using specimens from different body compartments. Sci Rep. 2014 July 11;4:5658.

138. Sharma SK, Kohli M, Chaubey J, Yadav RN, Sharma R, Singh BK, et al. Evaluation of Xpert MTB/RIF assay performance in diagnosing extrapulmonary tuberculosis among adults in a tertiary care centre in India. Eur Respir J. 2014 Oct;44(4):1090–3.

139. Mazzola E, Arosio M, Nava A, Fanti D, Gesu G, Farina C. Performance of real-time PCR Xpert ®MTB/RIF in diagnosing extrapulmonary tuberculosis. Infez Med. 2016 Dec 1;24(4):304–9.

140. Pang Y, Shang Y, Lu J, Liang Q, Dong L, Li Y, et al. GeneXpert MTB/RIF assay in the diagnosis of urinary tuberculosis from urine specimens. Sci Rep. 2017 July 21;7(1):6181.

141. Samuel BP, Michael JS, Chandrasingh J, Kumar S, Devasia A, Kekre NS. Efficacy and role of Xpert® Mycobacterium tuberculosis/rifampicin assay in urinary tuberculosis. Indian J Urol. 2018;34(4):268–72.

142. Habous M, Elimam MAE, Kumar R, Deesi ZAL. Evaluation of GeneXpert Mycobacterium tuberculosis/Rifampin for the Detection of Mycobacterium tuberculosis Complex and Rifampicin Resistance in Nonrespiratory Clinical Specimens. The International Journal of Mycobacteriology. 2019 June;8(2):132.

143. Chen Y, Wu P, Fu L, Liu Y hong, Zhang Y, Zhao Y. Multicentre evaluation of Xpert MTB/RIF assay in detecting urinary tract tuberculosis with urine samples. Sci Rep. 2019 July 30;9(1):11053.

144. Lopez AL, Aldaba JG, Morales-Dizon M, Sarol JN, Daag JV, Ama MaC, et al. Urine Xpert MTB/RIF for the diagnosis of childhood tuberculosis. International Journal of Infectious Diseases. 2019 Feb;79:44–6.

145. Mokaddas E, Ahmad S, Eldeen H. Performance Comparison of GeneXpert MTB/RIF and ProbeTec ET Tests for Rapid Molecular Diagnosis of Extrapulmonary Tuberculosis in a Low TB/MDR-TB Incidence Country. Med Princ Pract. 2021;30(3):277–84.

146. Song R, Click ES, McCarthy KD, Heilig CM, Mchembere W, Smith JP, et al. Sensitive and Feasible Specimen Collection and Testing Strategies for Diagnosing Tuberculosis in Young Children. JAMA Pediatr. 2021 May 1;175(5):e206069.

147. Liu P, Wang Y, Hao S, Qin Y. Comparison of the CapitalBio™Mycobacterium RT-PCR detection test and Xpert MTB/RIF assay for diagnosis of renal tuberculosis. Eur J Clin Microbiol Infect Dis. 2021 Mar;40(3):559–63.

148. Minnies S, Reeve BWP, Rockman L, Nyawo G, Naidoo CC, Kitchin N, et al. Xpert MTB/RIF Ultra Is Highly Sensitive for the Diagnosis of Tuberculosis Lymphadenitis in a High-HIV Setting. J Clin Microbiol. 2021 Nov 18;59(12):e0131621.

149. Wang Y, Tan J, Lei L, Yuan Y, Li W, Zhao Y, et al. The value of Xpert MTB/RIF assay of urine samples in the early diagnosis of smear-negative urinary tuberculosis. Eur J Med Res. 2022 Dec 20;27(1):300.

150. Moretó-Planas L, Mahajan R, Fidelle Nyikayo L, Ajack YBP, Tut Chol B, Osman E, et al. Xpert-Ultra Assay in Stool and Urine Samples to Improve Tuberculosis Diagnosis in Children: The Médecins Sans Frontières Experience in Guinea-Bissau and South Sudan. Open Forum Infect Dis. 2024 May;11(5):ofae221.

151. Dutschke A, Steiniche D, Jespersen S, Nanque JP, Medina C, Hønge BL, et al. Xpert MTB/RIF on urine samples to increase diagnosis of TB in people living with HIV in Guinea-Bissau. Int J Infect Dis. 2022 Nov;124 Suppl 1:S63–8.

152. Zahid QUA, Khursheed N, Adnan F, Zafar A. Concordance and discordance of GeneXpert MTB/RIF and conventional culture method for diagnosis of Extra-Pulmonary Tuberculosis at a tertiary care hospital in Pakistan. Pak J Med Sci. 2024 Jan;40(2ICON Suppl):S64–9.

153. Hueda-Zavaleta M, Gomez-de-la-Torre J, Barletta-Carrillo C, Flores-Flores C, Piscoche-Botello N, Miranda-Visalot C, et al. Diagnostic accuracy of Xpert MTB/RIF and Xpert ultra tests in pulmonary and extrapulmonary tuberculosis compared to Löwenstein-Jensen culture. Clinical Epidemiology and Global Health. 2024 Sept 1;29:101730.

154. Mitarai S, Oishi K, Fukasawa M, Yamashita H, Nagatake T, Matsumoto K. Clinical evaluation of polymerase chain reaction DNA amplification method for the diagnosis of pulmonary tuberculosis in patients with negative acid-fast bacilli smear. Tohoku J Exp Med. 1995 Sept;177(1):13–23.

155. Gamboa F, Manterola JM, Lonca J, Viñado B, Matas L, Giménez M, et al. Rapid detection of Mycobacterium tuberculosis in respiratory specimens, blood and other non-respiratory specimens by amplification of rRNA. Int J Tuberc Lung Dis. 1997 Dec;1(6):542–55.

156. Gamboa F, Dominguez J, Padilla E, Manterola JM, Gazapo E, Lonca J, et al. Rapid diagnosis of extrapulmonary tuberculosis by ligase chain reaction amplification. J Clin Microbiol. 1998 May;36(5):1324–9.

157. Aceti A, Zanetti S, Mura MS, Sechi LA, Turrini F, Saba F, et al. Identification of HIV patients with active pulmonary tuberculosis using urine based polymerase chain reaction assay. Thorax. 1999 Feb;54(2):145–6.

158. Hemal AK, Gupta NP, Rajeev TP, Kumar R, Dar L, Seth P. Polymerase chain reaction in clinically suspected genitourinary tuberculosis: comparison with intravenous urography, bladder biopsy, and urine acid fast bacilli culture. Urology. 2000 Oct 1;56(4):570–4.

159. Kafwabulula M, Ahmed K, Nagatake T, Gotoh J, Mitarai S, Oizumi K, et al. Evaluation of PCR-based methods for the diagnosis of tuberculosis by identification of mycobacterial DNA in urine samples. Int J Tuberc Lung Dis. 2002 Aug;6(8):732–7.

160. Marei AM, El-Behedy EM, Mohtady HA, Afify AF. Evaluation of a rapid bacteriophage-based method for the detection of Mycobacterium tuberculosis in clinical samples. J Med Microbiol. 2003 Apr;52(Pt 4):331–5.

161. Torrea G, Van de Perre P, Ouedraogo M, Zougba A, Sawadogo A, Dingtoumda B, et al. PCR-based detection of the Mycobacterium tuberculosis complex in urine of HIV-infected and uninfected pulmonary and extrapulmonary tuberculosis patients in Burkina Faso. J Med Microbiol. 2005 Jan;54(Pt 1):39–44.

162. Cannas A, Goletti D, Girardi E, Chiacchio T, Calvo L, Cuzzi G, et al. Mycobacterium tuberculosis DNA detection in soluble fraction of urine from pulmonary tuberculosis patients. Int J Tuberc Lung Dis. 2008 Feb;12(2):146–51.

163. Sener AG, Kurultay N, Afsar I. Evaluation of the results of Mycobacterium tuberculosis direct test (MTD) and Mycobacterial culture in urine samples. Braz J Microbiol. 2008 Oct;39(4):673–5.

164. García-Elorriaga G, Gracida-Osorno C, Carrillo-Montes G, González-Bonilla C. Clinical usefulness of the nested polymerase chain reaction in the diagnosis of extrapulmonary tuberculosis. Salud Publica Mex. 2009;51(3):240–5.

165. Gopinath K, Singh S. Urine as an adjunct specimen for the diagnosis of active pulmonary tuberculosis. Int J Infect Dis. 2009 May;13(3):374–9.

166. da Cruz HLA, de Albuquerque Montenegro R, de Araújo Lima JF, da Rocha Poroca D, da Costa Lima JF, Maria Lapa Montenegro L, et al. Evaluation of a nested-PCR for mycobacterium tuberculosis detection in blood and urine samples. Braz J Microbiol. 2011 Jan;42(1):321–9.

167. Ghaleb K, Afifi M, El-Gohary M. Assessment of diagnostic techniques of urinary tuberculosis. Mediterr J Hematol Infect Dis. 2013;5(1):e2013034.

168. Khan FUR, Cheema FA, Khan MU. Accuracy of Urinary PCR as Compared with Urine Culture for Early Diagnosis of Genitourinary Tuberculosis.

169. Kim JK, Bang WJ, Oh CY, Yoo C, Cho JS. Feasibility of the Interferon-γ Release Assay for the Diagnosis of Genitourinary Tuberculosis in an Endemic Area. Korean J Urol. 2013 Feb;54(2):123–6.

170. Heydari AA, Movahhede Danesh MR, Ghazvini K. Urine PCR evaluation to diagnose pulmonary tuberculosis. Jundishapur J Microbiol. 2014 Mar;7(3):e9311.

171. Lima JF da C, Guedes G de MR, Lima JF de A, Lira LA de S, Santos FCF, Arruda ME de, et al. Single-tube nested PCR assay with in-house DNA extraction for Mycobacterium tuberculosis detection in blood and urine. Rev Soc Bras Med Trop. 2015;48(6):731–8.

172. Jamshidi Makiani M, Davoodian P, Baghershiroodi M, Nejatizadeh AA, Fakkhar F, Zangeneh M, et al. Urine-Based Nested PCR for the Diagnosis of Mycobacterium tuberculosis: A Comparative Study Between HIV-Positive and HIV-Negative Patients. Jundishapur J Microbiol. 2016 Aug;9(8):e35634.

173. Ayatollahi J, Sheikhha MH, Abna Z, Tafti AD, Shahcheraghi SH. Evaluating nested-PCR of urine to diagnose smear positive pulmonary tuberculosis. Reviews and Research in Medical Microbiology. 2016 Oct;27(4):131.

174. Bordelon H, Ricks KM, Pask ME, Russ PK, Solinas F, Baglia ML, et al. Design and use of mouse control DNA for DNA biomarker extraction and PCR detection from urine: Application for transrenal Mycobacterium tuberculosis DNA detection. J Microbiol Methods. 2017 May;136:65–70.

175. Santos FCF, Lira LA de S, Montenegro R de A, Lima JF da C, Lima AS, Schindler HC, et al. Performance of the IS6110-TaqMan® assay in the diagnosis of extrapulmonary tuberculosis from different biological samples. Rev Soc Bras Med Trop. 2018;51(3):331–7.

176. Patel K, Nagel M, Wesolowski M, Dees S, Rivera-Milla E, Geldmacher C, et al. Evaluation of a Urine-Based Rapid Molecular Diagnostic Test with Potential to Be Used at Point-of-Care for Pulmonary Tuberculosis: Cape Town Cohort. J Mol Diagn. 2018 Mar;20(2):215–24.

177. Chemeda A, Abebe T, Ameni G, Worku A, Mihret A. Utility of urine as a clinical specimen for the diagnosis of pulmonary tuberculosis in people living with HIV in Addis Ababa, Ethiopia. J Clin Tuberc Other Mycobact Dis. 2019 Dec;17:100125.

178. Bisognin F, Lombardi G, Finelli C, Re MC, Dal Monte P. Simultaneous detection of Mycobacterium tuberculosis complex and resistance to Rifampicin and Isoniazid by MDR/MTB ELITe MGB® Kit for the diagnosis of tuberculosis. PLoS One. 2020;15(5):e0232632.

179. Costa-Lima JF da, Pimentel LMLM, Santos FCF, Salazar MP, Duarte RS, Mello FC de Q, et al. Rapid detection of Mycobacterium tuberculosis in children using blood and urine specimens. Rev Soc Bras Med Trop. 2020;53:e20200051.

180. Oreskovic A, Panpradist N, Marangu D, Ngwane MW, Magcaba ZP, Ngcobo S, et al. Diagnosing Pulmonary Tuberculosis by Using Sequence-Specific Purification of Urine Cell-Free DNA. J Clin Microbiol. 2021 July 19;59(8):e0007421.

181. Chang A, Mzava O, Djomnang LAK, Lenz JS, Burnham P, Kaplinsky P, et al. Metagenomic DNA sequencing to quantify Mycobacterium tuberculosis DNA and diagnose tuberculosis. Sci Rep. 2022 Oct 10;12(1):16972.

182. Kamra E, Alam D, Singh V, Kumar M, Chauhan M, Mehta PK. Diagnosis of urogenital tuberculosis by multiplex-nested PCR targeting mpt64 (Rv1980c) and IS6110: comparison with multiplex PCR and GeneXpert® MTB/RIF. Lett Appl Microbiol. 2022 Oct;75(4):857–68.

183. Araújo RM, Montenegro R de A, Peixoto ADS, Silva LL de S, da Costa RMPJ, Carvalho-Silva WHV, et al. Performance of IS6110-LAMP assay for detection of Mycobacterium tuberculosis complex in blood and urine samples from patients with extrapulmonary tuberculosis. Tuberculosis (Edinb). 2023 Dec;143:102423.

184. Tschan Y, Sasamalo M, Hiza H, Fellay J, Gagneux S, Reither K, et al. Diagnostic accuracy of a sequence-specific Mtb-DNA hybridization assay in urine: a case-control study including subclinical TB cases. Microbiology Spectrum. 2024 May 8;12(6):e00426-24.

185. Salazar MP, da Costa Lima Suassuna Monteiro JF, Veloso Carvalho-Silva WH, Nunes Diniz GT, Werkhauser RP, Lapa Montenegro LM, et al. Development and evaluation of a single-tube nested PCR with colorimetric assay for Mycobacterium tuberculosis detection. BioTechniques. 2024 June 24;76(6):235–44.

186. Sun L, Yuan Q, Feng JM, Yang CM, Yao L, Fan QL, et al. Rapid diagnosis in early stage renal tuberculosis by real-time polymerase chain reaction on renal biopsy specimens. Int J Tuberc Lung Dis. 2010 Mar;14(3):341–6.

187. Zhao N, Sun JY, Xu HP, Sun FY. Early Diagnosis of Tuberculosis-Associated IgA Nephropathy with ESAT-6. Tohoku J Exp Med. 2017 Apr;241(4):271–9.

188. Schön T, Gebre N, Sundqvist T, Aderaye G, Britton S. Effects of HIV co-infection and chemotherapy on the urinary levels of nitric oxide metabolites in patients with pulmonary tuberculosis. Scand J Infect Dis. 1999;31(2):123–6.

189. Yuksekol I, Ozkan M, Akgul O, Tozkoparan E, Al-Rashed M, Balkan A, et al. Urinary neopterin measurement as a non-invasive diagnostic method in pulmonary tuberculosis. Int J Tuberc Lung Dis. 2003 Aug;7(8):771–6.

190. Young BL, Mlamla Z, Gqamana PP, Smit S, Roberts T, Peter J, et al. The identification of tuberculosis biomarkers in human urine samples. Eur Respir J. 2014 June;43(6):1719–29.

191. Petrone L, Cannas A, Aloi F, Nsubuga M, Sserumkuma J, Nazziwa RA, et al. Blood or Urine IP-10 Cannot Discriminate between Active Tuberculosis and Respiratory Diseases Different from Tuberculosis in Children. Biomed Res Int. 2015;2015:589471.

192. Lim SH, Martino R, Anikst V, Xu Z, Mix S, Benjamin R, et al. Rapid Diagnosis of Tuberculosis from Analysis of Urine Volatile Organic Compounds. ACS Sens. 2016 July 22;1(7):852–6.

193. Sandlund J, Lim S, Queralto N, Huang R, Yun J, Taba B, et al. Development of colorimetric sensor array for diagnosis of tuberculosis through detection of urinary volatile organic compounds. Diagn Microbiol Infect Dis. 2018 Dec;92(4):299–304.

194. Wang J, Zhu X, Xiong X, Ge P, Liu H, Ren N, et al. Identification of potential urine proteins and microRNA biomarkers for the diagnosis of pulmonary tuberculosis patients. Emerg Microbes Infect. 2018 Apr 11;7(1):63.

195. Isa F, Collins S, Lee MH, Decome D, Dorvil N, Joseph P, et al. Mass Spectrometric Identification of Urinary Biomarkers of Pulmonary Tuberculosis. EBioMedicine. 2018 May;31:157–65.

196. Kim SY, Kim J, Kim DR, Kang YA, Bong S, Lee J, et al. Urine IP-10 as a biomarker of therapeutic response in patients with active pulmonary tuberculosis. BMC Infect Dis. 2018 May 29;18(1):240.

197. Fitzgerald BL, Islam MN, Graham B, Mahapatra S, Webb K, Boom WH, et al. Elucidation of a Human Urine Metabolite as a Seryl-Leucine Glycopeptide and as a Biomarker of Effective Anti-Tuberculosis Therapy. ACS Infect Dis. 2019 Mar 8;5(3):353–64.

198. Petrone L, Bondet V, Vanini V, Cuzzi G, Palmieri F, Palucci I, et al. First description of agonist and antagonist IP-10 in urine of patients with active TB. Int J Infect Dis. 2019 Jan;78:15–21.

199. Izquierdo-Garcia JL, Comella-Del-Barrio P, Campos-Olivas R, Villar-Hernández R, Prat-Aymerich C, De Souza-Galvão ML, et al. Discovery and validation of an NMR-based metabolomic profile in urine as TB biomarker. Sci Rep. 2020 Dec 18;10(1):22317.

200. Eribo OA, Leqheka MS, Malherbe ST, McAnda S, Stanley K, van der Spuy GD, et al. Host urine immunological biomarkers as potential candidates for the diagnosis of tuberculosis. Int J Infect Dis. 2020 Oct;99:473–81.

201. Deng J, Liu L, Yang Q, Wei C, Zhang H, Xin H, et al. Urinary metabolomic analysis to identify potential markers for the diagnosis of tuberculosis and latent tuberculosis. Archives of Biochemistry and Biophysics. 2021 June 15;704:108876.

202. Liu L, Deng J, Yang Q, Wei C, Liu B, Zhang H, et al. Urinary proteomic analysis to identify a potential protein biomarker panel for the diagnosis of tuberculosis. IUBMB Life. 2021 Aug;73(8):1073–83.

203. Comella-Del-Barrio P, Izquierdo-Garcia JL, Gautier J, Doresca MJC, Campos-Olivas R, Santiveri CM, et al. Urine NMR-based TB metabolic fingerprinting for the diagnosis of TB in children. Sci Rep. 2021 June 7;11(1):12006.

204. Elvira D. Association of Urinary Interferon Gamma Protein-10 Levels and Low Levels of Cluster of Differentiation 4 Serum in Patients with Tuberculosis-Human Immunodeficiency Virus Coinfection. Open Access Maced J Med Sci. 2021 Sept 7;9(A):707–10.

205. Kaushik A, Bandyopadhyay S, Porwal C, Srinivasan A, Rukmangadachar LA, Hariprasad G, et al. 2D-DIGE based urinary proteomics and functional enrichment studies to reveal novel Mycobacterium tuberculosis and human protein biomarker candidates for pulmonary tuberculosis. Biochem Biophys Res Commun. 2022 Sept 3;619:15–21.

206. Lyu L, Jia H, Liu Q, Ma W, Li Z, Pan L, et al. Individualized lipid profile in urine-derived extracellular vesicles from clinical patients with Mycobacterium tuberculosis infections. Front Microbiol. 2024;15:1409552.

207. Olivier C, Luies L. Metabolic insights into HIV/TB co-infection: an untargeted urinary metabolomics approach. Metabolomics. 2024 July 16;20(4):78.

208. Isaiah S, Loots DT, van Furth AMT, Davoren E, van Elsland S, Solomons R, et al. Urinary markers of Mycobacterium tuberculosis and dysbiosis in paediatric tuberculous meningitis cases undergoing treatment. Gut Pathog. 2024 Mar 12;16(1):14.
